# Stakeholders’ perspectives on communication gaps in cervical cancer prevention among women of Turkish, Moroccan and Syrian origin: a focus group study in the Netherlands

**DOI:** 10.1136/bmjopen-2026-120419

**Published:** 2026-10-20

**Authors:** Tharsini Veeravagu, Nora Hamdiui, Mart L Stein, Nadien H S Al-Rubaie, Josina L I Van Wijk, Martine P A Bouman, Rik Crutzen, Aura Timen

**Affiliations:** 1 Center for Infectious Disease Control (CIb), National Institute for Public Health and the Environment, Bilthoven, Netherlands; 2 Department of Primary and Community Care, Radboud University Medical Center, Nijmegen, Netherlands; 3 Department of Public and Occupational Health, Amsterdam University Medical Centres, Amsterdam, Netherlands; 4 Health Behaviours and Chronic Diseases, Amsterdam Public Health Research Institute, Amsterdam, Netherlands; 5 Center for Media and Health, Gouda, Netherlands; 6 Department of Health Promotion, Maastricht University School for Public Health and Primary Care, Maastricht, Netherlands

**Keywords:** Uterine Cervical Neoplasms, HPV Vaccine, Decision Making, Community-Based Participatory Research

## Abstract

**Objective:**

To gather stakeholders’ perspectives on the gaps between the information needs of women of Turkish, Moroccan and Syrian origin and the current communication practices regarding cervical cancer (CC) prevention programmes.

**Design:**

This qualitative study employed five focus group discussions (FGDs) with three categories of stakeholders. The social ecological model (SEM) guided the selection of stakeholders at the interpersonal, community and system level and the interpretation of findings.

**Setting:**

Data were collected in the Netherlands between January and February 2024.

**Participants:**

(1) Two FGDs with key community figures (n=20, interpersonal and community level), (2) two FGDs with healthcare professionals (n=12, interpersonal and system level) and (3) one FGD with communication experts, programme managers and policy makers (n=8, system level). Discussions with key community figures focused on cultural, religious, emotional and practical aspects of CC prevention programmes among women of Turkish, Moroccan and Syrian origin. Healthcare professionals discussed barriers and facilitators in communication practices, and the communication experts, programme managers and policy makers discussed the feasibility of suggested interventions by the first two stakeholder groups. The FGDs were transcribed verbatim in Dutch and thematically analysed.

**Results:**

The analysis resulted in six themes: (1) stakeholders’ perceptions on the considerations that women of Turkish, Moroccan and Syrian origin have when seeking, understanding and applying information about CC and CC prevention programmes; (2) current approach for health communication practices; (3) suggestions for interventions focused on women of Turkish, Moroccan and Syrian origin; (4) suggestions for interventions focused on the target population’s network; (5) variations among women of Turkish, Moroccan and Syrian origin that need to be considered and (6) conditions that the interventions should meet. Stakeholders described substantial gaps between women’s information needs and current communication practices, mainly related to misconceptions, limited awareness and doubts about the effectiveness of the CC prevention programmes. They characterised existing communication as predominantly top-down, from stakeholders at the system level to women at the individual level. Therefore, stakeholders emphasised the need to involve community-level stakeholders and to strengthen their communication skills and capacities to better support informed decision-making (IDM).

**Conclusions:**

Current CC prevention communication is perceived as predominantly top-down, from system level stakeholders to women at the individual level. Stakeholders highlighted the necessity of shifting towards dynamic interactions among stakeholders across all levels of the SEM. Partnerships between system-level and community-level stakeholders are recommended to enhance knowledge exchange and awareness of culturally relevant barriers. Collaborative approaches, such as joint educational sessions, co-creating information materials and interactive events, may improve recognisability, accessibility and trust in CC prevention programmes. These findings offer valuable insights into the feasibility of tailored interventions and the roles of various stakeholders, supporting IDM regarding CC prevention programmes among women of Turkish, Moroccan and Syrian origin.

**STRENGTHS AND LIMITATIONS OF THIS STUDY**

- The study design may have limited observations of interactions and potential synergies between different stakeholder categories and the exploration of deeper mechanisms of underlying stakeholders’ perceptions.
- Recruitment through project networks and subsequent snowball sampling may have favoured relatively engaged stakeholders, although our sample also included stakeholders without prior community engagement experience and none of the participants had prior information about the proposed interventions.
- Conducting separate focus group discussions per stakeholder category enabled an in-depth exploration of category-specific perspectives and experiences.
- During analysis, data from all stakeholder categories were combined to identify overarching patterns and insights that may support transferability to similar contexts.

## Background

 Cervical cancer (CC) is one of the dominant types of cancer diagnosed in women worldwide.^1^ CC is caused by an infection with the human papilloma virus (HPV).^2^ Worldwide, 604 196 women are diagnosed with CC each year and 279 583 die from the disease annually.^3^ In the Netherlands, 852 women are diagnosed with CC each year, 295 die from the disease annually and the 5-year prevalence of CC is 3.4%.^4^ To reduce this burden, CC screening and HPV vaccination programmes are implemented. The Dutch national CC screening programme has been implemented for women aged 30 to 60 years old, in the form of an HPV-based screening (detection of the presence of HPV).^5^ If HPV is found to be present, a cytology-based screening follows. Since 2010, HPV vaccination has been implemented in the Netherlands, initially only for 13-year-old girls. As of 2022, boys are also invited, and the age of invitation has been lowered to 10 years for both girls and boys. Although these CC prevention programmes are offered free of charge, participation rates remain low, especially among women with a migration background.^6–8^ These women show both low participation rates in CC prevention programmes and limited levels of informed decision-making (IDM).^9^ IDM is defined in several ways, with certain key aspects recurring in all these definitions. In the current study, we will adhere to the definition of IDM that follows two studies on cancer screening, highlighting the following key aspects: decisions based on sufficient knowledge, while being aware of and able to weigh all the positive and negative aspects involved, with a result that is in line with the decision maker’s values.^10 11^ Another study on cancer screening also argues that the focus for IDM is on transparent, balanced and objective risk communication, as it supports individual autonomy.^12^ Furthermore, voluntariness is also described as a key aspect of IDM, in the context of participation in population-based cancer screening.^11^

The Dutch National Institute of Public Health and the Environment (RIVM) has been taking several steps to improve the health communication practices in terms of accessibility and understandability to support IDM regarding CC prevention programmes for women with a migration background. For example, translations of online information materials were improved (https://www.rivm.nl/en/cervical-cancer-screening-programme/information-in-multiple-languages) and a platform with animated videos in simple Dutch was launched (https://bevolkingsonderzoek.steffie.nl/nl/modules/module_baarmoederhalskanker/). Furthermore, they adjusted the invitation letters by simplifying the language level and using more pictograms. Previously developed culturally sensitive educational videos are also implemented on the website of the RIVM^13^(https://www.rivm.nl/en/cervical-cancer-screening-programme/information-in-multiple-languages). Unfortunately, an interview study among women of Turkish, Moroccan and Syrian origin indicated that these adjusted and newly developed materials are often not easy to find.^14^

The prior conducted interview study among women of Turkish, Moroccan and Syrian origin showed that general practitioners (GPs) and key community figures are their most important sources of information regarding CC prevention.^14^ This study also indicated that IDM regarding CC prevention programmes is embedded in their social relationships and broader social contexts, rather than being an isolated individual process. These findings align with the social ecological model (SEM), stating that health behaviours and decisions are shaped by the interactions between factors at the individual, interpersonal, community and the system level.^15^ The individual level is focused on factors such as knowledge, attitudes and competencies. The interpersonal level refers to the social networks, including direct interaction with family, friends and others. The community level is related to the social and physical environment. The system level includes factors such as law, policy and cultural norms and values that affect health behaviour.^15^ To our knowledge, no studies in the Netherlands have specifically examined stakeholders’ perspectives on tailoring the communication practices for CC prevention programmes to the information needs of women of Turkish, Moroccan and Syrian origin. Understanding such multilevel stakeholder perceptions is helpful in identifying communication gaps and to develop feasible, tailored interventions that support IDM in these groups. The current study investigated the perspectives of stakeholders operating at different levels of the SEM. Considering perceptions from these different levels helps to identify the gaps between women’s information needs and current communication practices. The SEM also helps to outline multilevel approaches for tailoring health communication and how stakeholders at different levels of the SEM could be involved in interventions to support IDM regarding CC prevention programmes. The aim of this study was to identify what stakeholders perceive as gaps between the information needs of women of Turkish, Moroccan and Syrian origin and the current communication practices regarding CC prevention programmes. Furthermore, this study aimed to gather stakeholders’ perspectives on how to bridge these gaps. This will provide more insights into the feasibility of interventions aimed at improving health communication practices regarding CC prevention programmes and the potential role of stakeholders therein. Tailoring these health communication practices will support IDM regarding CC prevention programmes among women of Turkish, Moroccan and Syrian origin.

The research questions (RQs) are:

- What gaps do stakeholders identify between the information needs of women of Turkish, Moroccan and Syrian origin and the current communication practices regarding CC prevention programmes in the Netherlands?
- How do stakeholders see their own role in interventions to bridge these gaps and to what extent are these interventions feasible?
- Which conditions do stakeholders indicate as important for interventions aiming to bridge these gaps?

## Methods

### Study design

Focus group discussions (FGDs) were conducted in January and February 2024 to gather stakeholders’ perspectives on the gaps between the information needs of women of Turkish, Moroccan and Syrian origin and the current communication practices regarding CC screening and HPV vaccination. Furthermore, during each FGD the perspectives of stakeholders on how to bridge these gaps were discussed regarding both the CC screening programme and the HPV vaccination programme. FGDs are a useful method for discussing the potential suitability and feasibility of interventions,^16^ as they encourage participants to collaboratively explore and clarify their knowledge, attitudes, feelings, beliefs and experiences through interaction with others. The reporting of this study is in adherence to the Consolidated criteria for Reporting Qualitative research (COREQ) checklist.^17^ This study was guided by the SEM by informing the stakeholder selection and the interpretation of the results.

### Study population

We conducted five separate FGDs with diverse categories of stakeholders from different levels of the SEM: (1) two FGDs with key community figures (interpersonal and community level), (2) two FGDs with healthcare professionals (interpersonal and system level) and (3) one FGD with communication experts, policy makers and programme managers (system level). We discussed the cultural, religious, emotional and practical aspects regarding CC screening and HPV vaccination with the key community figures, based on previously conducted needs assessments.^9 14 18^ These key community figures are part of the Turkish, Moroccan and/or Syrian community themselves and therefore able to reflect on these aspects from their own experiences. Healthcare professionals discussed the barriers and facilitating factors they encounter in their function in communication practices regarding CC prevention to women of Turkish, Moroccan and Syrian origin. Based on the suggestions made by key community figures and healthcare professionals, we discussed the feasibility of the implementation of these suggested interventions with community experts, policy makers and programme managers. The FGDs were held separately with each category of stakeholders to keep the focus on their own areas of expertise.

### Recruitment

We used purposive sampling to recruit stakeholders who were directly involved in or influential for communication about CC prevention programmes among women of Turkish, Moroccan and Syrian origin. This approach was chosen, as it allowed us to capture a broad range of relevant perceptions on communication gaps and potential interventions from stakeholders operating at different levels of the SEM. We started the recruitment by reaching out to the network of the project team and their colleagues. We contacted them by email and telephone. This network consisted of volunteers at community centres and community organisations and healthcare professionals having patients of Turkish, Moroccan or Syrian origin. We also contacted other community organisations, community centres and healthcare centres outside the project team’s network via email and telephone. We asked them for individuals within their organisations who might be interested in participating. Furthermore, we placed posts on LinkedIn and on several websites and (digital) newspapers for healthcare professionals (eg, the website of the Dutch Association of Medical Assistants (in Dutch: *Nederlandse Vereniging van Doktersassistenten; nvda.nl*) and the digital newspaper of the National Immunisation Programme (in Dutch: *Rijksvaccinatieprogramma; RVP Nieuws*)). For participants in the FGD with community experts, policy makers and programme managers, we directly contacted the individuals working for the cancer screening organisation (‘Bevolkingsonderzoek Nederland’). Snowball sampling was conducted to recruit extra participants aiming for a small to medium-sized focus group of 5–8 participants per FGD, in line with commonly recommended group sizes for qualitative focus group studies.^19 20^ Individuals who indicated to be interested were informed about the study via a (digital) information letter. After confirmation of participation, we asked for their availability and planned the FGDs with at least five participants each. Recruitment continued until data saturation was reached within each stakeholder category, defined as the point at which no new themes emerged during the analysis of the last FGD

### Data collection

FGDs ranged from 5 to 14 participants. The largest group resulted from joining an existing, regularly meeting group of key community figures, and we chose not to exclude members of this group. During all FGDs, the moderators actively encouraged contributions from quieter participants and used targeted probing to ensure that multiple voices were heard. The FGD guide was constructed based on a prior conducted needs-assessment, which included a literature review^18^ and an interview study among women of Turkish, Moroccan, and Syrian origin.^14^ Initially, this resulted in an extensive list of questions and was transformed into several scenarios, to which participants could respond. The project team considered this scenario-based approach as more useful than the previous list of questions, as it allowed participants to engage with realistic and potentially familiar situations and encouraged them to reflect on their own experiences and perspectives. To test the duration, set up and design of this FGD guide, we conducted one pilot FGD with eight women of Turkish, Moroccan and Syrian origin, face-to-face in a local community centre. The pilot FGD lasted approximately 2 hours. Subsequently, we made some changes to the FGD guide, in terms of order of the discussion parts (see final set up of the guideline below under ‘FGD guide’ and in online supplementary material 1). The FGDs were held online via MS Teams, offline in a mosque and in a meeting room at the RIVM. Two of the researchers, a female moderator and a female assistant (TV and NHSA-R), facilitated the FGDs. Both hold a Master’s degree and have previous experience with conducting qualitative research and moderating FGDs. NHSA-R had a good command of Modern Standard-Arabic and shared similar cultural and religious backgrounds with women of Turkish, Moroccan and Syrian origin. This facilitated trust and openness in the FGDs with key community figures. At the same time, the research team engaged in continuous reflexivity regarding how shared background and assumptions might shape questioning, probing and interpretation during the FGDs and subsequent data analysis. Each FGD was spoken in Dutch and took approximately 2 hours. Prior to the start of the FGDs, participants were asked to fill in a short questionnaire (see online supplementary material 2). Here, we asked them about their job/function, years of work experience and the first two digits of their postal code. These questions were important to gain insight into the professional backgrounds and geographical distribution of the stakeholders, which could influence their perspectives obtained during the FGDs. This questionnaire also included the informed consent for participating in this study (see online supplementary material 3).

#### FGD guide

We started the FGDs with a brainstorm based on several scenarios. Here, we discussed communication practices regarding CC screening and/or HPV vaccination in certain situations. We asked for suggestions of interventions that could support women of Turkish, Moroccan and Syrian origin in making an informed decision regarding participation. In the second part, we discussed a few statements with potential IDM-supporting interventions that were based on the findings of a previously conducted interview study.^14^ This interview study investigated the information needs and preferences of women of Turkish, Moroccan and Syrian origin regarding CC prevention programmes. Furthermore, we asked key community figures and healthcare professionals how they see their role in these potential interventions. These potential interventions were also based on the findings of the previous interview study with the target population.^14^ In the final part of the FGD, we also asked participants to reflect on the findings of this previous interview study. This was done at the end of the FGD to avoid influencing participants’ ideas and suggestions during the prior brainstorming and discussion phases, thereby encouraging them to reflect on the findings of this previous interview study, based on their own perspectives and experiences. This approach was chosen to strengthen the validity of the findings by minimising potential bias and ensuring that the suggestions for interventions reflected participants’ authentic perspectives (see online supplemental material 1).

### Data analysis

The FGDs were audio-recorded and transcribed verbatim. The female assistant (NHSA-R) also took field notes during the FGDs. The transcripts were complemented by the notes to verify what was said by whom during the FGDs. The FGDs were analysed using the software program MAXQDA V.2024.1. We performed an inductive thematic analysis, starting with one of the researchers (TV) conducting open coding (labelling the data by breaking the data down into discrete parts), followed by axial coding (systematic comparison of the coded text) to analyse the FGDs.^21^ Subsequently, themes and subthemes were generated (selective coding). Three out of the five FGDs were double coded by a second researcher (NHSA-R) to ensure the reliability of the analysis. Discrepancies were discussed in the presence of a third researcher (NH) until consensus was reached. For a comprehensive assessment of overall trends and patterns, we combined data from the three stakeholder categories to identify overarching themes rather than stakeholder-specific themes. Within each overarching theme, we explicitly distinguished and described similarities and differences between the stakeholder categories so that divergent perceptions were preserved and presented.

### Patient and public involvement

This study directly involved the target populations through qualitative methods. As part of the broader project, we first conducted a needs assessment in which women of Turkish, Moroccan and Syrian origin were interviewed about their information needs and preferences regarding CC prevention programmes.^14^ Their priorities and experiences informed the selection of relevant stakeholder categories for the present FGDs. Women who participated in the needs assessment were not explicitly involved in the formal design of the FGD study protocol or in recruitment procedures. However, to assess the burden and time required for participation, we conducted a pilot FGD with a group of women of Turkish, Moroccan, and Syrian origin. The findings from the needs assessment and the present study were subsequently used to develop interventions to support IDM regarding CC prevention programmes with women of Turkish, Moroccan, and Syrian origin (a training for key community figures and an online video intervention). We involved women of Turkish, Moroccan, and Syrian origin in the development, implementation and evaluation of these interventions.

## Results

### Participants characteristics

In total, we held two FGDs with key community figures, two FGDs with healthcare professionals and one FGD with communication experts, policy makers and programme managers. Three out of five FGDs were held online via MS Teams; the other two were conducted face-to-face at a suitable location preferred by the participants. The number of participants per FGD varied from 5 to 14. Table 1 provides an overview of the participants per FGD, including their gender and a description of their job/function.

**Table 1:** Overview of participants per FGD

Stakeholder category	Participant	Gender	Job/function
Healthcare professionals 1 (online)	1	Man	General practitioner
	2	Woman	Youth nurse, project employee
	3	Woman	Community leader, volunteer in community centre, general practitioner assistant
	4	Woman	Youth healthcare employee
	5	Woman	General practitioner assistant
	6	Woman	Youth healthcare professional
	7	Woman	Nurse sexual health
Key community figure 1 (online)	8	Woman	Teacher and mentor
	9	Woman	Volunteer in mosque
	10	Woman	Community leader
	11	Woman	Employee at foundation/organisation
	12	Woman	Volunteer in community centre
	13	Woman	Volunteer in community centre
Healthcare professionals 2 (online)	14	Man	Doctor, project leader vaccination uptake
	15	Woman	General practitioner assistant
	16	Man	Medical advisor, formerly doctor society and health in youth healthcare
	17	Man	Medical advisor (formerly general practitioner)
	18	Man	Medical advisor (formerly general practitioner)
Key community figure 2 (face-to-face at a mosque)	19	Woman	Volunteer in mosque
	20	Woman	Volunteer in mosque
	21	Woman	Community leader
	22	Woman	Volunteer in mosque
	23	Woman	Volunteer in mosque
	24	Woman	Volunteer in mosque
	25	Woman	Volunteer in mosque
	26	Woman	Community leader
	27	Woman	Volunteer in mosque
	28	Woman	Volunteer in mosque
	29	Woman	Volunteer in mosque
	30	Woman	Volunteer in mosque
	31	Woman	Volunteer in mosque
	32	Woman	Volunteer in mosque
Communication experts, policy makers and programme managers (face-to-face at the RIVM office)	33	Woman	Doctor society and health, medical advisor
	34	Woman	Policy maker
	35	Woman	Programme manager
	36	Woman	Communication expert
	37	Woman	Communication expert
	38	Woman	Programme employee
	39	Woman	Programme manager
	40	Woman	Programme manager

FGD, focus group discussion; RIVM, National Institute for Public Health and the Environment.

### Thematic analysis

During the FGDs, stakeholders shared their reflections on and experiences with health communication practices to women of Turkish, Moroccan and/or Syrian origin. Through interactions between stakeholders, the feasibility of IDM-supporting interventions and how stakeholders view their own role therein were also discussed. Six themes were generated in the thematic analysis: (1) stakeholders’ perspectives on the considerations that women of Turkish, Moroccan and Syrian origin have when seeking, understanding and applying information about CC and CC prevention programmes; (2) current approach for health communication practices; (3) suggestions for interventions focused on women of Turkish, Moroccan and Syrian origin; (4) suggestions for interventions focused on the target population’s network; (5) variations among women of Turkish, Moroccan and Syrian origin that need to be considered and (6) conditions that the interventions should meet. In the following sections, the RQs were answered by describing the findings per theme. These themes were described following three phases: the current situation, the desired situation and considerations for bridging the gap.

## The current situation according to stakeholders

We started by describing the current situation of the communication practices regarding CC prevention programmes. The stakeholders’ perspectives and experiences are described to answer RQ A: What gaps do stakeholders identify between the information needs of women of Turkish, Moroccan and Syrian origin and the current communication practices regarding CC prevention programmes?

### Theme 1: stakeholders’ perspectives on the considerations that women of Turkish, Moroccan and Syrian origin have when seeking, understanding and applying information about CC and CC prevention programmes

Key community figures indicated a misinterpretation at the interpersonal level of the SEM, namely that women of Turkish, Moroccan and Syrian origin might think that they receive an invitation letter for CC screening because their GP thinks that they are sick. Hereby, key community figures pointed to a gap in the GP–patient relationship. According to the key community figures, this reflects limited awareness of the procedures of the regular invitation programmes operating at system level.

I reassured her [referring to her mother] that this is a routine examination, commonly conducted at this age, and that participation does not imply the presence of any health problems. The invitation to participate is not based on individual concerns, but on age group eligibility.

- Employee at foundation/organization

For the HPV vaccination, key community figures indicated to hear a lot of doubts regarding its safety and effectiveness. This was also indicated by healthcare professionals. Both stakeholder categories mentioned that women of Turkish, Moroccan, and Syrian origin think that this vaccination is not necessary or that many women are sceptical regarding the safety of the vaccine. Key community figures and healthcare professionals also mentioned that women of Turkish, Moroccan, and Syrian origin have more trust in the old-trusted vaccines, rather than the somewhat ‘newer’ HPV vaccination.

You often see that they stick to the ‘old-trusted’ vaccines for child diseases. But for the HPV vaccination, it is something ‘new’, something scary.

- Volunteer in a mosque

The main consideration in seeking information about CC mentioned by key community figures was that this should not be discussed with men. At this point, key community figures reflected factors at the community level by addressing shared cultural and religious norms and values regarding sexuality.

It is culture-bound that a girl is more likely to reach out to her mother rather than her father with these kinds of concerns, due to feelings of embarrassment. Such topics are discussed less frequently with fathers. When it comes to menstruation, for example, girls tend to reach out to their mothers.

- Volunteer in a mosque

### Theme 2: current approach for health communication practices

This theme described which practices are currently used for the communication regarding CC prevention. Stakeholders shared their experiences with the existing information materials for women of Turkish, Moroccan, and Syrian origin and the barriers they face. They also introduced techniques that they use themselves and the challenges they deal with while using the current available information materials.

#### Theme 2A: existing information materials for women of Turkish, Moroccan and Syrian origin

Communication experts, policy makers and programme managers shared their experiences with a telephone information line. They indicated that such a telephone line had been implemented at the system level in several health programmes (eg, COVID-19 vaccination and colorectal cancer screening), but it was rarely used in practice at the individual level. Stakeholders in this FGD also pointed out that collaborations with the community level were more often done, for example, working with community organisations and providing education at schools. One of the healthcare professionals indicated that information about CC screening is part of the Dutch civil integration course for individuals with a migration background. However, the key community figures indicated that this might not be the right moment to expose women with a migration background to these types of topics, as they have other priorities to learn at that time. Besides these approaches, communication experts, policy makers, and programme managers indicated that social media was used in previous attempts to spread information regarding CC prevention.

#### Theme 2B: barriers for women of Turkish, Moroccan and Syrian origin in receiving information

Despite the existing information materials, stakeholders across all categories mentioned that women of Turkish, Moroccan, and Syrian origin still experience barriers in obtaining, processing, and understanding information regarding CC screening and HPV vaccination. As indicated by both key community figures and healthcare professionals, the information is often not sufficiently clear and raises questions.

This topic raises questions, as it concerns one’s own body. The HPV vaccination introduces something unfamiliar to the body, and procedures such as CC screening can also prompt uncertainty. It is natural for women to be left with questions or concerns after reading about this*.*

- Teacher and mentor

They also emphasised that in the first place, women of Turkish, Moroccan and Syrian origin should be willing to receive information regarding CC screening. Therefore, key community figures indicated that they should be aware of the severity of the disease, which is not always the case. Many healthcare professionals described the pressure of the social norm, embedded in the community level, among women of Turkish, Moroccan, and Syrian origin as a barrier for obtaining, processing, and understanding information. For example, they described that these women experience peer pressure and blindly copy what their friends and/or family members do and say, reflecting factors at the interpersonal level. One of the healthcare professionals indicated that this could also facilitate the spread of misinformation. According to stakeholders across all categories, another barrier experienced by women of Turkish, Moroccan, and Syrian origin is seeking care from a healthcare professional, such as their GP, referring to interactions at the interpersonal level. Stakeholders across all categories identified a lowered proficiency of the Dutch language as an underlying cause at the individual level for this barrier, especially for the older women with a migration background. Healthcare professionals also mentioned lower health prioritisation as a barrier at the individual level, particularly for younger women with a migration background. Healthcare professionals described these younger women as a ‘forgotten group’ in health communication practices.

Precisely the group between 20 and 40 years, whom we want to target with information regarding CC screening, is a group we actually rarely encounter in practice. This is likely due to the relatively low incidence of disease in this age category and the limited opportunities for structured health communication practices, as there are few routine interactions with them. As a result, they may generally be considered as a ‘forgotten group’*.*

- GP

Another barrier among the younger women with a migration background, as mentioned by the stakeholders, probably comes from a lack of time and feeling more comfortable by using the internet to gather information. For the older women with a migration background, stakeholders across all categories mentioned that the barrier for visiting their GP is often because of experiencing a language barrier at the individual level and the discomfort they experience at community level due to shame and taboo regarding speaking about their intimate parts related to CC screening.

In my experience, we mainly saw women of Turkish origin in our general practice, who consulted us for a wide variety of health issues. However, when the conversation shifted towards gynecological issues […], they often appeared noticeably more uncomfortable.

- Medical advisor (formerly GP)

#### Theme 2C: current and previous approach of stakeholders

In the FGDs with healthcare professionals, we also spoke about their practices in health communication to their patients at the interpersonal level. Most of them indicated to use a demand-driven approach. For example, they ask their clients what they already know, what they want to know, and what they are afraid of. This was also something that was confirmed during the last FGD with communication experts, policy makers, and programme managers. Key community figures indicated that they use a neutral tone of voice when communicating information. They mentioned that they do not give any advice, but stick to factual medical information. If someone directly asks for personal advice, key community figures indicated that they often refer to the GP or a Youth Healthcare Professional.

#### Theme 2D: barriers for healthcare professionals in health communication practices

Although healthcare professionals also try their best to meet the information needs of women of Turkish, Moroccan, and Syrian origin with their demand-driven approach, they indicated to still encounter barriers in health communication practices. The main reason for this, as indicated during the FGDs, was a lack of time during their consultation hours. This occurs from a restricted consultation time determined at system level leading to constraints at communication at the interpersonal level. There is also a gap in that women of Turkish, Moroccan and Syrian origin identified their GP as their preferred source of information regarding both CC screening and HPV vaccination, whereas GPs described that at the system level it is defined that the GP works in a symptoms-oriented manner, meaning that patients do not visit their GP on a regular basis to discuss certain matters including prevention, but only when they have certain symptoms. Also, in the current used medical record systems, healthcare professionals indicated that it is hard to identify if their patient has already participated in the CC screening and/or if their child(ren) is/are vaccinated against HPV. This is due to the fact that only a general ‘yes/no’ indicator for preventive care is recorded in the patient’s electronic patient dossier (EPD). This is without specifying which prevention programmes (e.g. one of the cancer screening programmes or vaccination programmes) are involved. To overcome this (technical) barrier, healthcare professionals suggested enhancing the EPD to provide more detailed information, enabling them to identify in which particular preventive care programmes their patients are participating, and in which not.

Normally, if someone has participated in population screening, it will be linked to ‘preventive care’ in the EPD. Maybe it would be useful if we could directly see that this is regarding CC screening, for example, and separately for breast cancer screening as well. Now you only see ‘preventive care’, but that can be anything.

- GP assistant

#### Theme 2E: existing support for healthcare professionals

A few healthcare professionals mentioned that they followed e-learnings and education courses to overcome barriers in health communication practices with women with a migration background and other vulnerable groups. They indicated that these materials primarily focus on interpersonal interactions related to preventive care programmes, motivational conversations, and non-vaccinating individuals in general, but do not address group-specific information needs at community level.

## The desired situation according to stakeholders

In the next part, we explored stakeholders’ perspectives on the feasibility of potential interventions aimed to support IDM regarding CC prevention programmes and discussed whether they could play a role in it or not. Theme 3 described interventions focused on the individual, and theme 4 described interventions focused on the (social) network. The results from these themes are used to answer RQ B: How do stakeholders see their own role in these interventions and to what extent are these interventions feasible?

### Theme 3: suggestions for interventions focused on women of Turkish, Moroccan and Syrian origin

This theme described the interventions focusing on women of Turkish, Moroccan and Syrian origin that were suggested during the FGDs. Stakeholders stated that there is a need for interventions that are specifically targeted at the individual and interpersonal level.

#### Theme 3A: organise informational meetings

The intervention most frequently mentioned by stakeholders across all categories was the organisation of informational meetings, with a particular emphasis on those targeting older women with a migration background. Stakeholders across all categories mentioned that these informational meetings should be organised at the interpersonal level, via collaborations with key community leaders and healthcare professionals. Key community leaders, who are highly trusted at the community level, reported having valuable insight into culturally sensitive methods for health communication practices. Healthcare professionals were identified as trusted sources of information at the interpersonal level. Both stakeholder categories acknowledged the potential for mutual collaboration. Stakeholders across all categories suggested to emphasise the fact that Islam highly prioritises taking good care of your own body and health, reflecting a facilitator at community level. Communication experts, policy makers, and programme managers suggested also linking a participation-action to the informational meetings. For example, provide support for making an appointment for the HPV vaccination for those who want to vaccinate their children after attending the informational meeting. For the CC screening, it was mentioned that it could be helpful to support women with requesting the self-testing toolkit. Stakeholders indicated that requesting the toolkit is often experienced as complicated at the individual level due to the needed digital skills. However, communication experts also stated that the main purpose during such informational meetings should be to communicate information and not to persuade participation. Therefore, stakeholders emphasised that the tone during informational meetings should remain neutral.

#### Theme 3B: short videos on social media

For younger women with a migration background stakeholder across all categories highly recommended the use of audiovisual materials via social media as health communication practice. For example, short videos with women from Turkish, Moroccan and Syrian origin sharing their experiences and thoughts on CC screening and HPV vaccination. Key community figures expressed that it is necessary to share both positive and negative views, to keep the information neutral. For younger women with a migration background who are born and raised in the Netherlands, stakeholders mentioned that these videos could be in Dutch. They noted that these younger women with a migration background experience minimal language barriers.

Wouldn’t it work to have someone from the target population tell something about it in short videos. So that instead of flyers going around, videos will circulate. If you have someone who reaches the target population, and shares his or her experiences with the vaccine and/or the CC screening within like 30 seconds? I think when you hear and see it in different ways, you become more open to it.

- Teacher and mentor

### Theme 4: suggestions for the interventions focused on the target population’s social network

Besides interventions focused on the target population, stakeholders also suggested interventions focused on their social network. The focus was on stakeholders at the interpersonal level, namely key community figures and healthcare professionals, who were identified as important sources of information by women of Turkish, Moroccan and Syrian origin in a previous study.^14^

#### Theme 4A: training for key community figures

To organise informational meetings, key community figures indicated the need to increase their knowledge regarding CC and its prevention programmes. Stakeholders across all categories suggested that this could be done via capacity building at the community level by providing training, since many of the key community figures do not have experience with communicating information regarding CC prevention. Key community figures mentioned that the training should be focused on equipping them with comprehensive knowledge regarding CC, CC screening and/or HPV vaccination. In addition, they also stated that they want to gain practical experience in communicating information to women within their communities, for instance through role-playing exercises during the training. Key community figures emphasised the importance of ensuring that these training sessions are accessible, for example by organising them locally within each region. Furthermore, they suggested that both the training sessions and the informational meetings should be made appealing and engaging. Stakeholders identified elements such as food and music as potential connecting factors that could promote participation in training and cohesion during the informational meetings.

As I do myself, every three months I organize an informational meeting, including food and music. This approach helps to bring women together, and then at least 80 women attend these meetings*.*

- Key community figure, GP assistant

#### Theme 4B: support for healthcare professionals

Healthcare professionals indicated that they also have a need for further training at the interpersonal level, particularly in conversational techniques. They indicated that this could also be done in the form of role playing, but due to limited time, more flexible interventions would be better fitting for them. An example given was informative podcasts or e-learnings which they can go through at their preferred moment. Stakeholders indicated that healthcare professionals also could benefit from visual tools in the consultation room, which they can use as conversation openers. Thereby, stakeholders indicated that asking patients about their previous participation during consultation hours can also be valuable, because this gives women the room to ask any further questions.

How worthfully would it be if a GP asks: have you ever participated before? That is the only question that should be asked. And if the person comes up with a follow-up question, then you have an opening to the conversation*.*

- Communication expert

## Considerations for bridging the gap according to the stakeholders

Lastly, we described the required changes in health communication practices according to the stakeholders. Themes 5 and 6 described the results that are linked to RQ C: Which conditions do stakeholders indicate as important for interventions aiming to bridge these gaps?

### Theme 5: variations among women of Turkish, Moroccan and Syrian origin that need to be considered

Stakeholders across all categories emphasised the similarities at community level regarding cultural and religious aspects among women of Turkish, Moroccan, and Syrian origin. For example, the Islamic religion and its associated norms and values regarding sexuality, virginity, and fertility. However, during the FGDs, stakeholders also highlighted that it is important to consider the differences among women of Turkish, Moroccan, and Syrian origin.

#### Theme 5A: religious variety

First, stakeholders across all categories indicated that it is important to acknowledge the religious variety among women of Turkish, Moroccan, and Syrian origin. In addition, they emphasised that there is also a part of the population from Syrian origin that adheres to Christianity. Moreover, participants explained that within the Islamic religion itself various movements exist, reflecting further religious variation. They mentioned that these variations in religious perspectives also lead to a variety in norms and values regarding sexuality, virginity, and fertility. Therefore, stakeholders across all categories emphasised the need to consider these various perspectives in communication practices regarding CC prevention programmes. They also noted that, despite these differences, there are notable similarities in religious perspectives and practices. During the FGD with communication experts, policy makers, and programme managers, one of the stakeholders highlighted that many of the information needs regarding sexuality, virginity and fertility expressed by women of Turkish, Moroccan, and Syrian origin are similar to those of a 29/34-year-old woman in the general public. Stakeholders across all categories acknowledged that these aspects should be embedded in health communication practices to better adhere to the information needs of women of Turkish, Moroccan, and Syrian origin, as well as other young Dutch women without a migration background.

The things that emerge from here [referring to the interview study^14^ ] are not very different from our investigation among 29–34-year-olds. They have somewhat the same questions*.*

- Communication expert

#### Theme 5B: varying levels of language proficiency

According to the stakeholders across all categories, another aspect at the individual level that needs to be considered is the variety in language proficiency among women of Turkish, Moroccan, and Syrian origin. Stakeholders indicated that these women may experience language barriers. Key community figures and healthcare professionals reported that older women with a migration background often rely on their children or other family members to translate (medical) information. To address this barrier, stakeholders across all categories suggested that information materials should also be communicated in their relevant native languages, along with the invitation letter. Furthermore, stakeholders highlighted that a substantial proportion of these women with a migration background is illiterate, meaning that some individuals are unable to read or write, either in Dutch or in their native language. The influence of educational levels was also noted by stakeholders. Key community figures indicated that those with higher levels of education might be more critical of the provided information, while healthcare professionals observed that decision-making among less educated individuals is often more strongly influenced at the interpersonal level via their social environment.

#### Theme 5C: variations between generations

According to key community figures, the older women with a migration background are more reluctant to actively seek information compared with younger women with a migration background. Key community figures suggested that this reluctance may explain the lower levels of awareness and knowledge regarding CC and its prevention programmes among older women with a migration background. In contrast, younger women with a migration background were described as more critical of the information provided. Key community figures indicated that younger women with a migration background often possess more advanced digital skills and, as a result, may be more inclined to actively seek information compared with older women with a migration background.

From what I see, there is a clear difference between age categories. I think that the youth is more tending to actively seek information themselves, whereas the older women with a migration background are more likely to wait until information is provided to them. For example, I often notice that my parents prefer to wait for an official letter or ask me to find the information for them, making it as easy as possible. In contrast, people of my own age are much more proactive in searching for information themselves*.*

- Teacher and mentor

#### Theme 5D: variations between urban and rural settings

A final aspect described by stakeholders across all categories that should be considered in health communication practices to women of Turkish, Moroccan, and Syrian origin were the variations between women with a migration background living in urban versus rural settings. According to key community figures, women with a migration background living in urban settings tend to be more critical and are generally more open to receiving information. They noted that in urban settings, there are often more mosques and community organisations that actively organise activities to share information at community level. In contrast, they mentioned that such opportunities are often more limited in rural settings. Key community figures mentioned that the board of mosques often have contact with other mosques in the same region. They suggested that it would be helpful to jointly organise activities to share information in the rural settings of the Netherlands.

### Theme 6: conditions that the interventions should meet

This theme described the conditions that interventions, aiming at improving IDM for women of Turkish, Moroccan and Syrian origin, should meet, according to the stakeholders across all categories. These conditions described with whom, where, when and how the information needs of women of Turkish, Moroccan and Syrian origin should be met.

#### Theme 6A: health communication practices via the social network (who)

Stakeholders across all categories emphasised that health communication practices should be done at the interpersonal and community level via social networks. Therefore, they suggested collaborating with key community figures, healthcare professionals and experts-by-experience from the target population. Key community figures recommended collaborating with mosque boards or the imam (an Islamic religious leader), as these individuals are trusted and well-known at community level. Their established relationships enable successful health communication practices and address concerns or reduce fears. The type of healthcare professionals that were mentioned to provide information on CC screening and HPV vaccination were GPs, GP assistants and youth healthcare workers. Both healthcare professionals and communication experts, policy makers and programme managers indicated that midwives are also willing to play a role in this. As these stakeholders explained, midwives see women at a logical time to start the conversation regarding CC screening and HPV vaccination. Healthcare professionals and communication experts, policy makers and programme managers indicated that most women presenting to midwives are at the eligible age for CC screening and can therefore be reminded to consider participation in CC screening after pregnancy. Furthermore, healthcare professionals and communication experts, policy makers and programme managers mentioned that it may also be that those women have children who will be or already have been invited for the HPV vaccination. Stakeholders across all categories indicated that working with experts-by-experience enables sharing stories from the target population. In all FGDs, this was mentioned as a way to raise more awareness, since this will lead to more recognition and provide them with information that is in line with their way of living. According to stakeholders across all categories, this might lead to an increase of perceived susceptibility to and severity of CC.

#### Theme 6B: health communication practices at locations they are already visiting (where)

The overarching factor mentioned by stakeholders across all categories about where information regarding CC screening and HPV vaccination should be communicated, was to do this at locations that women of Turkish, Moroccan and Syrian origin already visit. These were locations at interpersonal and community level, for example mosques, schools, community centres and healthcare facilities. Other public places mentioned by stakeholders across all categories were libraries, (train) stations, sports clubs and indoor playgrounds.

#### Theme 6C: integrate with pre-existing community events (when)

Regarding optimal timing for communication practices regarding CC screening and HPV vaccination, stakeholders across all categories advocated for the integration of such information into pre-existing community events and routines. This approach was recommended by healthcare professionals on the grounds that many women are unlikely to actively seek information themselves. Furthermore, key community figures suggested that information could be communicated during the Friday Prayers at mosques, as these gatherings are already well-attended by women in the community. They explained that such an approach offers individuals the opportunity to share their worries and ask questions. To facilitate this, healthcare professionals indicated that it is first important to build trust, as this fosters appreciation and facilitates open communication. Both key community figures and healthcare professionals also noted that individuals want to feel that they are being taken seriously. Therefore, key community figures mentioned that receiving information from someone with a similar background is more likely to be trusted and adopted.

It is important that when a key community figure organizes informational meetings, to collaborate with a healthcare professional, preferably one who shares a similar migration or cultural background. This ensures that both the key community figure and the women attending the informational meeting feel taken seriously. If an unrelated individual would deliver the information, it might not be received in the same way*.*

- Volunteer in a mosque

#### Theme 6D: a combination of digital, written and verbal information (how)

Stakeholders across all categories indicated that it is very important to use a combination of digital and written information. They recommended using audiovisual materials on social media, because that will reach many individuals (especially the younger generations) and is easy to share within communities. However, they also stated that it is still necessary to also use written and verbal information which is communicated face-to-face. This is especially for older generations who experience more difficulties with accessing digital information. Furthermore, stakeholders across all categories emphasised the importance of investigating how to make the information findable for women of Turkish, Moroccan and Syrian origin. They mentioned that relevant alternatives for information materials, for example translations of the information brochures, are already made available from system level, but do not reach women with a migration background at the individual level adequately. Therefore, stakeholders recommended making these materials more findable. Key community figures recommended communicating translated versions of the informational brochures along with the invitation letters. However, during the FGD with communication experts, policy makers and programme managers, several stakeholders expressed concerns regarding the implementation of this strategy. Conversely, healthcare professionals indicated that the communication of translated informational brochures within the central areas of healthcare facilities would be a more feasible approach. Thereby, several stakeholders across all categories also highlighted the added value of presenting audiovisual information materials (eg, videos) on display screens in these central areas.

The translations for the HPV vaccination are quite impossible to find. So, if someone is already looking for information, but cannot find it, the job is to make it easier to find. Currently, you have to try very hard to obtain the information.

- Communication expert

## Discussion

### Principal findings

This study explored stakeholders’ perspectives on current communication practices for CC prevention programmes. Stakeholders across all categories identified several gaps in these practices and suggested potential interventions to support IDM regarding CC prevention programmes. They mentioned key aspects necessary to consider for bridging these gaps. Stakeholders predominantly described communication gaps and potential interventions as relevant to women of Turkish, Moroccan and Syrian origin collectively, and did not propose distinct strategies for each group. This is consistent with our aim to explore communication approaches that could be implemented for these groups of women. At the same time, stakeholders pointed to important differences that should be considered when tailoring communication, such as religion, language proficiency, generation of migration and living in urban vs rural settings (see theme 5). Stakeholders across all categories largely converged in their descriptions of communication gaps and feasible interventions. Divergent or minority perceptions did not emerge and therefore did not form distinct themes, suggesting a shared perception of the main barriers and needs. According to the stakeholders’ perceptions, the identified gaps might be attributed to misconceptions, lack of awareness and doubts regarding the programmes’ effectiveness. Therefore, stakeholders across all categories highly recommend to also include stakeholders from the interpersonal and community level in health communication practices, rather than operating solely from the system level. Key community leaders and healthcare professionals indicated to experience a lack of support within the current health communication infrastructure or from public health authorities in tailoring health communication practices to the information needs of women of Turkish, Moroccan and Syrian origin. Therefore, they highlighted the need for interventions aimed at enhancing their skills in communication practices regarding CC prevention to the needs of women of Turkish, Moroccan and Syrian origin. Strengthening these stakeholders’ competencies might support women of Turkish, Moroccan and Syrian origin in making an informed decision regarding participation in CC prevention programmes.

### Involvement of stakeholders from community level in health communication practices

While the SEM was our primary conceptual framework to capture multi-level influences on IDM, specific health behaviour theories, including the COM-B model (Capability-Opportunity-Motivation-Behaviour),^22^ the Social Cognitive Theory^23^ and the Reasoned Action Approach,^24^ emphasise that IDM depends not only on individuals’ knowledge and their practical capabilities at the individual level, but also on personal motivation and self-efficacy. On the interpersonal level, social support also plays a role, indicating that in the context of CC prevention programmes IDM can be understood as a multi-level process in which the individual and interpersonal level are interacting. Findings from the present study indicate that, according to stakeholders’ perceptions, social support is insufficiently considered in current health communication practices. Role definitions in primary care are determined at system level, for example, that the GPs should work in a symptoms-oriented focus. This does not align with the needs and expectations of women of Turkish, Moroccan and Syrian origin who preferred their GP also for preventative health. Stakeholders indicated that the interpersonal and community level are often not actively involved, although they could play a key role in supporting IDM. A study conducted in Flanders (Belgium), which explored the perspectives of health and social professionals regarding barriers and facilitators to CC screening among women with lower participation rates, including women with a migration background, also highlighted the need for involvement of stakeholders operating at the interpersonal and community level (such as trusted key community figures) in communication practices regarding CC prevention programmes.^25^

### Interventions with cultural, religious and emotional resonance

Women of Turkish, Moroccan and Syrian origin indicated a need to receive information about the practical, emotional, religious and cultural aspects regarding CC prevention.^9 18^ Stakeholders in this study also described their perceptions on how to better align health communication practices with the lived experiences of these women. One approach suggested was the use of narratives from experts-by-experience, which is in line with the Narrative Communication Theory.^26^ A randomised controlled trial (RCT) among college women in the USA used this theory in a four-armed intervention RCT.^27^ Participants were randomly assigned to either a peer-only video-intervention, an experts-only video-intervention, a combined peer-intervention and experts’ video-intervention, or a control group. The results of that study showed that the combined video-intervention resulted in a higher increase of self-efficacy and intention regarding HPV vaccination uptake.^27^ This suggests that sharing experiences, additional to factual medical information, may positively influence attitudes towards and greater trust in healthcare prevention programmes.

### Strengths and limitations of the study

This study presented some limitations. For example, conducting only separate FGDs may have limited opportunities to observe interactions and potential synergies between different stakeholder categories. An additional mixed focus group could have provided further insight into how integration and collaboration between key community figures might be achieved in practice. Such a setting might have illuminated pathways for more enhanced stakeholder engagement and intersectoral cooperation. Nonetheless, mixing stakeholder categories in FGDs carries the risk that participants may feel less comfortable expressing their views, particularly when discussing sensitive topics or when power dynamics are at play. This could have constrained the openness of the discussions and potentially hindered the collection of genuine and comprehensive insights from all stakeholder categories. During the analysis, we combined data from all stakeholder categories. This was done to identify overarching patterns and insights that may support transferability to similar contexts, but may have limited the observation of category-specific phenomena.

Another limitation of this study is that, although the thematic analysis was comprehensive, several themes remained largely intervention-oriented, in line with our focus group design and practical aims. The study design limited the exploration of deeper mechanisms underlying stakeholders’ perceptions. Future studies using individual in-depth interviews with stakeholders could help to explain why stakeholders hold these perceptions and further investigate the rationales and assumptions behind their suggestions for interventions. Our recruitment strategy, which relied on project networks and subsequent snowball sampling, may have favoured the inclusion of stakeholders who were relatively engaged in promotion activities. However, our sample also included stakeholders without prior experience in health promotion activities at community level, and participants were not aware of the specific proposed interventions beforehand. This reduced the likelihood that pre-existing positive attitudes towards the interventions themselves biased recruitment.

Besides those limitations, a key strength of this study is the focus on exploring the perspectives of diverse stakeholders, particularly those operating at the community level. Interpersonal and community level stakeholders play a pivotal role in reaching women of Turkish, Moroccan and Syrian origin, as they can interact in their own social networks. For example, key community figures share similar cultural and religious norms and values and if they are actively involved in communication practices regarding CC prevention, these aspects could be adequately incorporated. Moreover, women of Turkish, Moroccan and Syrian origin identified these stakeholders as gatekeepers for the information needs and concerns within their communities,^14^ making their involvement highly valuable for the development of culturally sensitive and contextually appropriate interventions. It is, therefore, lucrative to investigate their perspectives and provide them with the appropriate tools to support their efforts in increasing IDM in CC prevention programmes.

Another strength is the decision to conduct separate FGDs for each stakeholder category. This approach allowed for an exploration of the specific perspectives and experiences within each stakeholder category. The homogeneity of the groups fostered a safe environment, which encouraged participants to share openly and facilitated rich, detailed discussions.

### Conclusion and implications for future practice

This study reveals stakeholders’ perspectives on the gaps between the information needs of women of Turkish, Moroccan and Syrian origin and the current communication practices regarding CC prevention programmes. According to the stakeholders, current health communication practices remain predominantly top-down, from system level to individual level, with insufficiently being tailored to the variations within the target population and their lived experiences. Stakeholders see an important role for themselves in bridging these gaps. They identified the need for a transfer towards interactive health communication practices. To achieve this, stakeholders suggest establishing partnerships between healthcare professionals (interpersonal and system level) and key community figures, religious leaders and teachers (interpersonal and community level). Stakeholders suggested that healthcare professionals could support key community figures by increasing their knowledge regarding CC and CC prevention programmes. The other way around, key community figures could provide healthcare professionals and other stakeholders from the system level with valuable insights into the barriers individuals face in making informed decisions regarding CC prevention, as well as highlight the cultural, religious, emotional and practical aspects associated with these barriers. They could support familiarity by making health communication practices more recognisable and accessible, and fostering trust between individuals and the CC prevention programmes. For example, this can be done by organising collaborative educational sessions for key community figures and healthcare professionals, co-creation of information materials, and holding interactive events where women of Turkish, Moroccan and Syrian origin could share their lived experiences, concerns and ask questions. Stakeholders expressed the need for considering the variations among women of Turkish, Moroccan and Syrian origin. Moreover, for successful implementation, they mentioned that it is recommended to align the interventions as closely as possible with the lived experiences of women of Turkish, Moroccan and Syrian origin. Our findings informed the development of a training for key community figures and culturally sensitive videos regarding CC screening. The videos were evaluated in a qualitative and quantitative study.

## Supplementary material

10.1136/bmjopen-2026-120419 online supplemental file 1

10.1136/bmjopen-2026-120419 online supplemental file 2

10.1136/bmjopen-2026-120419 online supplemental file 3

## Data Availability

Data are available on reasonable request.
