# Supplementary material for: Stakeholders’ perspectives on communication gaps in cervical cancer prevention among women of Turkish, Moroccan and Syrian origin: a focus group study in the Netherlands
Source: BMJ Open. 2026 Oct 20;16(10):e120419. doi: 10.1136/bmjopen-2026-120419 (PMC13684154; doi:10.1136/bmjopen-2026-120419)
Supplement: Supplementary file 1 [file bmjopen-2026-120419.reviewer_comments.pdf]

PEER REVIEW HISTORY

BMJ Open publishes all reviews undertaken for accepted manuscripts. Reviewers are asked to complete a checklist review form and are provided with free text boxes to elaborate on their assessment. These free text comments are reproduced below.

ARTICLE DETAILS

Title (Provisional)

Stakeholders' perspectives on communication gaps in cervical cancer prevention among women of Turkish, Moroccan, and Syrian origin: a focus group study in the Netherlands

Authors

Veeravagu, Tharsini; Hamdiui, Nora; Stein, Mart L; Al-Rubaie, Nadien H.S.; Van Wijk, Josina L.I.; Bouman, Martine P.A.; Crutzen, Rik; Timen, Aura

---

VERSION 1 - REVIEW

**Reviewer**	**1**
**Name**	**Destaw, Alemnew**
**Affiliation**	**Mizan-Tepi University, Public health**
**Date**	**29-Jul-2026**
**COI**	**None**

Thank you for the opportunity to review the manuscript entitled ***"Identifying gaps and stakeholders' perspectives in communication practices regarding cervical cancer prevention among Turkish-, Moroccan-, and Syrian-Dutch women: a focus group study."*** The study addresses an important public health question and represents a well-conducted qualitative investigation with substantial effort invested in data collection and presentation. The topic is timely and relevant, and the findings have potential implications for improving culturally tailored communication strategies.

Nevertheless, the manuscript would benefit from revision before publication. The principal concerns relate to methodological reporting (particularly within the Methods section), the analytical depth of the qualitative findings, the distinction between stakeholder perspectives and participants' actual experiences, and some over interpretation within the Discussion and Conclusion. These issues appear addressable through revision and do not undermine the overall value of the study.

Title:

- ✓ Consider revising the title to refer to the study as a qualitative study (or qualitative focus group study), as focus group discussions are primarily a qualitative data collection method rather than the overall research design.

Abstract:

- ✓ The abstract specifies the number of focus group discussions but does not report the number of participants included overall or within each stakeholder group. Including the sample size would improve transparency and allow readers to better assess the breadth of stakeholder representation.
- ✓ The Results section primarily lists the six themes identified but provides very limited information regarding the substantive findings within those themes. Including one or two key findings illustrating the major communication gaps and recommended interventions would considerably strengthen the abstract and better reflect the study's contribution.
- ✓ The abstract repeatedly refers to the Socio-Ecological Model (SEM), yet it remains unclear how this framework guided the study design, participant selection, data analysis, or interpretation of findings. Clarifying the role of SEM would improve methodological transparency.
- ✓ The sentence "Data collection is conducted in the Netherlands..." should be revised to the past tense (e.g., "Data were collected..."), as the study has already been completed.
- ✓ The Conclusions introduce the statement that current communication is predominantly "top-down," yet this key finding is not clearly reflected in the Results section of the abstract. Consider ensuring that the principal conclusions are directly supported by the summarized findings presented in the Results.

Background:

- ✓ The opening paragraph would be strengthened by incorporating the most recent global and Dutch cervical cancer epidemiological estimates (e.g., the latest GLOBOCAN/IARC data). Presenting the current disease burden before introducing prevention programmes would better justify the public health importance of the study.
- ✓ The introductory section could be organised more clearly by separating the epidemiological burden of cervical cancer, current prevention strategies, the persistent problem of low participation among migrant women, and the resulting research gap. Such restructuring would improve readability and strengthen the rationale for the study.
- ✓ The manuscript currently uses a mixture of citation styles. Please ensure consistency according to the journal's referencing guidelines.

- ✓ Although the authors acknowledge that informed decision-making has multiple definitions, they primarily adopt a definition originating from prenatal screening research. The rationale for selecting this framework over definitions developed specifically for cancer screening or vaccination contexts should be clarified.
- ✓ The Socio-Ecological Model is introduced as the conceptual framework underpinning the study; however, the background does not clearly explain how this framework informed the study design, stakeholder selection, data collection, or planned analysis. Strengthening the theoretical justification for using SEM would improve the conceptual foundation of the study.
- ✓ The statement that "there are no studies" appears rather absolute. Please clarify whether this statement refers to the Netherlands, Europe, or the broader international literature.

Method:

- ✓ The manuscript states that "this study used an inductive study design." Please clarify the study design and ensure it is described consistently throughout the manuscript, as induction generally refers to the analytical approach rather than the research design.
- ✓ The manuscript does not explain how the final sample size was determined. Please clarify whether thematic saturation (or information power) was achieved and how the decision to stop recruitment after five focus groups was made.
- ✓ Although the recruitment process is described, the sampling strategy itself is not explicitly stated. Please clarify whether purposive, convenience, criterion, or another sampling approach was employed and explain why it was appropriate for the study objectives.
- ✓ Although the moderators are identified, the manuscript provides limited information regarding researcher reflexivity (e.g., professional background, qualitative research experience, relationship with participants, and potential influence on data collection and interpretation). Additional information would strengthen methodological transparency.
- ✓ The rationale for combining data from three distinct stakeholder groups into overarching themes requires further justification. While common themes are informative, presenting stakeholder-specific themes or a clearer explanation of how divergent perspectives were preserved would strengthen the analysis.

Results

- ✓ The thematic analysis is comprehensive; however, several themes remain largely descriptive and intervention-oriented rather than interpretive. Consider strengthening the analytical depth by identifying higher-order concepts that explain why stakeholders

hold these perspectives instead of primarily categorizing recommendations.

- ✓ Given that the Socio-Ecological Model is presented as the study's conceptual framework, the Results would be strengthened by explicitly relating the themes to the different SEM levels or illustrating how interactions across these levels emerged from the analysis.
- ✓ The findings frequently combine perspectives across stakeholder categories, making it difficult to determine whether particular views were shared by all groups or expressed by only one stakeholder category. Presenting areas of convergence and divergence more explicitly would strengthen the credibility and usefulness of the findings.
- ✓ The Results primarily emphasize areas of agreement among participants. Were any divergent or minority perspectives identified? Reporting contradictory viewpoints would provide a more balanced and nuanced interpretation.
- ✓ Some findings are presented collectively for Turkish-, Moroccan-, and Syrian-Dutch women despite acknowledging important differences between these populations later in the Results. Consider ensuring that conclusions remain appropriately nuanced and avoid unnecessary generalization across distinct migrant communities.
- ✓ The Results occasionally present practical recommendations alongside empirical findings. Consider separating participants' reported perspectives from the authors' broader recommendations, reserving interpretation and implications primarily for the Discussion section.

Discussion

- ✓ The Discussion occasionally presents stakeholders' perceptions as though they represent the actual experiences or needs of Turkish-, Moroccan-, and Syrian-Dutch women. The interpretation should consistently distinguish between stakeholders' views and evidence obtained directly from the target population. This distinction is important for maintaining interpretive validity.
- ✓ The Discussion occasionally implies causal or effectiveness claims regarding proposed interventions. Given the exploratory qualitative design, these statements should be tempered to reflect that the study identifies perceived opportunities rather than demonstrating intervention effectiveness.
- ✓ Throughout the manuscript, consider using terminology consistent with qualitative research methodology. For example, "transferability" may be more appropriate than "generalisability" when discussing the broader applicability of qualitative findings.
- ✓ Several behavioural theories (COM-B, Social Cognitive Theory, Reasoned Action Approach, Narrative Communication Theory) are introduced for the first time in the

Discussion. Their relationship with the Socio-Ecological Model, which serves as the study's primary conceptual framework, should be better explained to avoid conceptual fragmentation. Alternatively, if these theories were intended to inform the study, they should be introduced earlier in the Background and reflected consistently throughout the manuscript.

- ✓ The conclusion would be strengthened by focusing primarily on the study's principal findings and their implications. The description of ongoing intervention development is interesting but is not itself a finding of the present study and could be shortened or relocated.

Thank you!

Reviewer **2**

Name **Manga, Simon**

Affiliation **University of Alabama at Birmingham Health System, Obstetrics and Gynecology**

Date **10-Aug-2026**

COI **None**

This manuscript addresses an important and underexplored topic on improving communication around cervical cancer screening and HPV vaccination among migrant populations through engagement of community and healthcare stakeholders. The study is timely, relevant to public health, and provides potentially actionable insights for improving IDM. The inclusion of multiple stakeholder groups and use of the socio-ecological model are notable strengths. However, there are a few minor comments that the authors should consider addressing to further strengthen the manuscript:

1. The manuscript should address saturation and reflexivity
2. Participants recruitment through project networks and subsequently snowball sampling raises concerns of potential bias. This is because participants may already be highly engaged in health promotion activities, leading to favorable views toward community engagement interventions
3. The manuscript states FGDs ranged from 5 to 14 participants. A focus group of 14 participants is relatively large and may limit participation from quieter individuals.
4. A little more detail of the pilot FGD is required e.g. number of participants, Duration, Location
5. The moderators of the FGDs are identified only as female researchers (TV and NHS). Readers will be interested to know a little more about them e.g. academic qualification, specialty, experience

VERSION 1 - AUTHOR RESPONSE

Reviewer: 1
Mr. Alemnew Destaw, Mizan-Tepi University, Martin Luther University Halle Wittenberg

Comments to the Author:
Thank you for the opportunity to review the manuscript entitled "Identifying gaps and stakeholders' perspectives in communication practices regarding cervical cancer prevention among Turkish-, Moroccan-, and Syrian-Dutch women: a focus group study." The study addresses an important public health question and represents a well-conducted qualitative investigation with substantial effort invested in data collection and presentation. The topic is timely and relevant, and the findings have potential implications for improving culturally tailored communication strategies.
Nevertheless, the manuscript would benefit from revision before publication. The principal concerns relate to methodological reporting (particularly within the Methods section), the analytical depth of the qualitative findings, the distinction between stakeholder perspectives and participants' actual experiences, and some over interpretation within the Discussion and Conclusion. These issues appear addressable through revision and do not undermine the overall value of the study.

Title:

- Consider revising the title to refer to the study as a qualitative study (or qualitative focus group study), as focus group discussions are primarily a qualitative data collection method rather than the overall research design.

Amended. We revised the title *(r. 1-4 of the marked manuscript).* This is now stated as follows:

> *''Stakeholders' perspectives on communication gaps in cervical cancer prevention among women of Turkish, Moroccan, and Syrian origin: a focus group study in the Netherlands.''*

Abstract:

- The abstract specifies the number of focus group discussions but does not report the number of participants included overall or within each stakeholder group. Including the sample size would improve transparency and allow readers to better assess the breadth of stakeholder representation.

Amended. We have included the number of participants within each stakeholder group in the abstract *(r. 40-42 of manuscript incl. revisions).* This is now stated as follows:

> *''(1) Two FGDs with key community figures (n= 20, interpersonal and community level), (2) two FGDs with healthcare professionals (n=12, interpersonal and system level), and (3) one FGD with communication experts, program managers, and policy makers (n= 8, system level).''*

- The Results section primarily lists the six themes identified but provides very limited information regarding the substantive findings within those themes. Including one or two key findings illustrating the major communication gaps and recommended interventions would considerably strengthen the abstract and better reflect the study's contribution.

Amended. We have included additional description of the findings within the themes in the Results section of the abstract *(r. 56-62 of the marked manuscript).* This is stated as follows:

''Stakeholders described substantial gaps between women's information needs and current communication, mainly related to misconceptions, limited awareness, and doubts about the effectiveness of the CC prevention programs. They characterized existing communication as predominantly top-down, from stakeholders at the system level to women at the individual level. Therefore, stakeholders emphasized the need to involve community-level stakeholders and to strengthen their communication skills and capacities to better support informed decision-making (IDM).''

- The abstract repeatedly refers to the Socio-Ecological Model (SEM), yet it remains unclear how this framework guided the study design, participant selection, data analysis, or interpretation of findings. Clarifying the role of SEM would improve methodological transparency.

Amended. We have included further justification for using the SEM in the abstract *(r. 33-35 of the marked manuscript).* This is stated as follows:

'The Social-Ecological Model (SEM) guided the selection of stakeholders at the interpersonal, community, and system level and the interpretation of findings.''

- The sentence "Data collection is conducted in the Netherlands..." should be revised to the past tense (e.g., "Data were collected..."), as the study has already been completed.

Amended. We have revised the sentence to the past tense *(r. 37-38 of the marked manuscript).* This is now stated as follows:

*''**Setting:** Data were collected in the Netherlands between January and February 2024.''*

- The Conclusions introduce the statement that current communication is predominantly "top-down," yet this key finding is not clearly reflected in the Results section of the abstract. Consider ensuring that the principal conclusions are directly supported by the summarized findings presented in the Results.

Amended. We have revised the Results and Conclusions section of the abstract for a better alignment *(r. 56-69 of the marked manuscript).* This is now stated as follows:

''Stakeholders described substantial gaps between women's information needs and current communication, mainly related to misconceptions, limited awareness, and doubts about the effectiveness of the CC prevention programs. They characterized existing communication as predominantly top-down, from stakeholders at the system level to women at the individual level. Therefore, stakeholders emphasized the need to involve community-level stakeholders and to strengthen their communication skills and capacities to better support informed decision-making (IDM).

***Conclusions:** Current CC prevention communication is perceived as predominantly top-down, from system level stakeholders to women at the individual level.''*

<u>Background:</u>

- The opening paragraph would be strengthened by incorporating the most recent global and Dutch cervical cancer epidemiological estimates (e.g., the latest GLOBOCAN/IARC data).

Presenting the current disease burden before introducing prevention programmes would better justify the public health importance of the study.

Amended. We have included the global and Dutch CC incidence and mortality rates, and the Dutch prevalence rate in the first paragraph of the background section of the manuscript *(r. 109-113 of the marked manuscript)*. This is stated as follows:

> *''Worldwide, 604 196 women are diagnosed with CC each year and 279 583 die from the disease annually. In the Netherlands, 852 women are diagnosed with CC each year in the Netherlands, 295 die from the disease annually, and the 5-year prevalence of CC is 3,4% (4). To reduce this burden, CC screening and HPV vaccination programs are implemented.''*

- The introductory section could be organised more clearly by separating the epidemiological burden of cervical cancer, current prevention strategies, the persistent problem of low participation among migrant women, and the resulting research gap. Such restructuring would improve readability and strengthen the rationale for the study

Thank you for this suggestion. We have strengthened the opening paragraph by incorporating the incidence, mortality, and prevalence rates to address the epidemiological burden of cervical cancer (see previous point). After describing the current CC prevention strategies and the disparities among migrant women in terms of participation and IDM, we included a justification for the need of investigating stakeholders' perspectives guided by the Social-Ecological Model (SEM) *(r. 150-166 of the marked manuscript)*. This is stated as follows:

> *''The prior conducted interview study among women of Turkish, Moroccan, and Syrian origin showed that general practitioners (GPs) and key community figures are their most important sources of information regarding CC prevention (3). This study also indicated that IDM regarding CC prevention programs is embedded in their social relationships and broader social contexts, rather than being an isolated individual process. These findings align with the Social Ecological Model (SEM), stating that health behaviors and decisions are shaped by the interactions between factors at the individual, interpersonal, community, and the system level (5). The individual level is focused on factors such as knowledge, attitudes, and competencies. The interpersonal level refers to the social networks, including direct interaction with family, friends, and others. The community level is related to the social and physical environment. The system level includes factors such as law, policy, and cultural norms and values that affect health behavior (5). To our knowledge, no studies in the Netherlands have specifically examined stakeholders' perspectives on tailoring the communication practices for CC prevention programs to the information needs of women of Turkish, Moroccan, and Syrian origin. Understanding such multi-level stakeholder perspectives is helpful in identifying communication gaps and to develop feasible, tailored interventions that support IDM in these groups.''*

- The manuscript currently uses a mixture of citation styles. Please ensure consistency according to the journal's referencing guidelines.

We appreciated the attention to referencing consistency. We have carefully checked the manuscript against the journal's guidelines and can confirm that the Vancouver citation style is now used consistently throughout the manuscript. There was one sentence in the introduction that presented

the author-date format *(r. 125-126 of the marked manuscript)*. This is now converted to Vancouver style and is stated as follows:

> *''Another study on cancer screening also argues that the focus for IDM is on transparent, balanced, and objective risk communication, as it supports individual autonomy (6).'*

- Although the authors acknowledge that informed decision-making has multiple definitions, they primarily adopt a definition originating from prenatal screening research. The rationale for selecting this framework over definitions developed specifically for cancer screening or vaccination contexts should be clarified.

Thank you for addressing this point. The definition used in this study was based on several studies, including both studies on prenatal and cancer screening. However, to avoid confusion regarding the rationale of selecting this definition, we adjusted the references to studies on cancer screening only *(r. 121-128 of the marked manuscript)*. This is now stated as follows:

> *''In the current study, we will adhere to the definition of IDM that follows from two studies on cancer screening, highlighting the following key aspects: decisions based on sufficient knowledge, while being aware of and able to weigh all the positive and negative aspects involved, with a result that is in line with the decision maker's values (7). Another study on cancer screening also argues that the focus for IDM is on transparent, balanced, and objective risk communication, as it supports individual autonomy (6). Furthermore, voluntariness is also described as a key aspect of IDM, in the context of participation in population-based cancer screening (7).''*

- The Socio-Ecological Model is introduced as the conceptual framework underpinning the study; however, the background does not clearly explain how this framework informed the study design, stakeholder selection, data collection, or planned analysis. Strengthening the theoretical justification for using SEM would improve the conceptual foundation of the study.

Amended. We have included further justification for using the SEM in the background section of the manuscript *(r. 150-171 of the marked manuscript).* This is stated as follows:

> *''The prior conducted interview study among women of Turkish, Moroccan, and Syrian origin showed that general practitioners (GPs) and key community figures are their most important sources of information regarding CC prevention (3). This study also indicated that IDM regarding CC prevention programs is embedded in their social relationships and broader social contexts, rather than being an isolated individual process. These findings align with the Social Ecological Model (SEM), stating that health behaviors and decisions are shaped by the interactions between the individual, community, and the system level (5). The individual level is focused on factors such as knowledge, attitudes, and competencies. The interpersonal level refers to the social networks, including direct interaction with family, friends, and others. The community level is related to the social and physical environment. The system level includes factors such as law, policy, and cultural norms and values that affect health behavior (5). To our knowledge, no studies in the Netherlands have specifically examined stakeholders' perspectives on tailoring the communication practices for CC prevention programs to the information needs of women of Turkish, Moroccan, and Syrian origin. Understanding such multi-level stakeholder perspectives is helpful to identify communication gaps and to develop feasible, tailored interventions that support IDM in these groups. The current study investigated the perspectives of stakeholders operating at different levels of the SEM.*

Considering perspectives from these different levels helps to identify the gaps between women's information needs and current communication practices. The SEM also helps to outline multi-level approaches for tailoring health communication and how stakeholders at different levels of the SEM could be involved in interventions to support IDM regarding CC prevention programs.''

Similarly, we also included this justification in the methods section of the manuscript *(r. 199-200 of the marked manuscript)*. This is stated as follows:

''This study was guided by the SEM by informing the stakeholder selection and the interpretation of the results.''

- The statement that "there are no studies" appears rather absolute. Please clarify whether this statement refers to the Netherlands, Europe, or the broader international literature.

Amended. We have adjusted the statement by nuancing the wording *(r. 161-164 of the marked manuscript).* This is stated as follows:

''To our knowledge, no studies in the Netherlands have specifically examined stakeholders' perspectives on tailoring the communication practices for CC prevention programs to the information needs of women of Turkish, Moroccan, and Syrian origin.''

Method:

- The manuscript states that "this study used an inductive study design." Please clarify the study design and ensure it is described consistently throughout the manuscript, as induction generally refers to the analytical approach rather than the research design.

Amended. We have removed the sentence under the 'Study Design' part in the Methods section *(r. 200-202 of the marked manuscript).* Furthermore, we clarified the sentences under the 'Data Analysis' part in the Methods (*r. 299-302 of the marked manuscript).* This is now stated as follows:

''We performed an inductive thematic analysis, starting with one of the researchers (TV) conducting open coding (labelling the data by breaking the data down in discrete parts), followed by axial coding (systematic comparison of the coded text) to analyze the FGDs (8). Subsequently, themes and subthemes were generated (selective coding).''

- The manuscript does not explain how the final sample size was determined. Please clarify whether thematic saturation (or information power) was achieved and how the decision to stop recruitment after five focus groups was made.

Thank you for addressing this issue. We have included a sentence under the 'Recruitment' part in the Methods section explaining that the determination of our final sample size was based on thematic saturation *(r. 242-244 of the marked manuscript).* This is stated as follows:

''Recruitment continued until data saturation was reached within each stakeholder category, defined as the point at which no new themes emerged during the analysis of the last FGD''

- Although the recruitment process is described, the sampling strategy itself is not explicitly stated. Please clarify whether purposive, convenience, criterion, or another sampling approach was employed and explain why it was appropriate for the study objectives.

Thank you for addressing this issue. We have included a sentence under the 'Recruitment' part in the Methods section describing that we used purposive sampling as our sampling strategy *(r. 221-225 of the marked manuscript).* This is stated as follows:

> *''We used purposive sampling to recruit stakeholders who were directly involved in or influential for communication about CC prevention programs among women of Turkish, Moroccan, and Syrian origin. This approach was chosen, as it allowed us to capture a broad range of relevant perspectives on communication gaps and potential interventions from stakeholders operating at different levels of the SEM.''*

- Although the moderators are identified, the manuscript provides limited information regarding researcher reflexivity (e.g., professional background, qualitative research experience, relationship with participants, and potential influence on data collection and interpretation). Additional information would strengthen methodological transparency.

Thank you for addressing this issue. We have included a sentence under the 'Data Collection part in the Methods section with additional information regarding the professional background and qualitative research experience of the moderators *(r. 263-264 of the manuscript incl. revisions).* This is stated as follows:

> *''Both hold a Master's degree and have previous experience with conducting qualitative research and moderating FGDs.''*

- The rationale for combining data from three distinct stakeholder groups into overarching themes requires further justification. While common themes are informative, presenting stakeholder-specific themes or a clearer explanation of how divergent perspectives were preserved would strengthen the analysis.

Amended. We have clarified the justification for combining data into overarching themes *(r. 304-309 of the marked manuscript).* This is now stated as follows:

> *''For a comprehensive assessment of overall trends and patterns, we combined data from the three stakeholder categories to identify overarching themes, rather than stakeholder-specific themes. Within each overarching theme, we explicitly distinguished and described similarities and differences between the stakeholder categories, so that divergent perspectives were preserved and presented.''*

<u>Results</u>

- The thematic analysis is comprehensive; however, several themes remain largely descriptive and intervention-oriented rather than interpretive. Consider strengthening the analytical depth by identifying higher-order concepts that explain why stakeholders hold these perspectives instead of primarily categorizing recommendations.

Thank you for addressing this issue. We acknowledge that several themes are predominantly intervention-oriented, reflecting the main goal of the study: to identify stakeholders' perspectives on communication gaps and feasible strategies to address them. Our focus group design limited the extent to which we could explore the underlying reasons why stakeholders hold these specific perspectives. We have therefore added this point as a limitation in the Discussion section and recommended that future research using individual in-depth interviews with stakeholders could strengthen the analytical depth regarding stakeholders' rationales and assumptions behind their suggestions *(r. 835-840 of the marked manuscript).*

''Another limitation of this study is that, although the thematic analysis was comprehensive, several themes remained largely intervention-oriented, in line with our focus group design and practical aims. The study design limited the exploration of deeper mechanisms underlying stakeholders' perspectives. Future studies using individual in-depth interviews with stakeholders could help to explain why stakeholders hold these perspectives and further investigate the rationales and assumptions behind their suggestions for interventions.''

- Given that the Socio-Ecological Model is presented as the study's conceptual framework, the Results would be strengthened by explicitly relating the themes to the different SEM levels or illustrating how interactions across these levels emerged from the analysis.

Thank you for this suggestion. We have revised the Results section of the manuscript by relating the identified themes to the corresponding level of the SEM. In Theme 1, this is now stated as follows:

''Key community figures indicated a misinterpretation at the interpersonal level of the SEM, namely that women of Turkish, Moroccan, and Syrian origin might think that they receive an invitation letter for CC screening, because their GP thinks that they are sick. Hereby, key community figures pointed to a gap in the GP-patient relationship. According to the key community figures, this reflects limited awareness of the procedures of the regular invitation programs operating at system level.'' - (r. 359-364 of the marked manuscript)

''The main consideration in seeking information about CC mentioned by key community figures was that this should not be discussed with men. At this point, key community figures reflected factors at the community level, by addressing shared cultural and religious norms and values regarding sexuality.'' (r. 380-383 of the marked manuscript)

Theme 2 is now stated as follows:

''Communication experts, policy makers, and program managers shared their experiences with a telephone information line. They indicated that such a telephone line had been implemented at the system level in several health programs (e.g. Covid-19 vaccination and colorectal cancer screening), but it was rarely used in practice at the individual level. Stakeholders in this FGD also pointed out that collaborations with the community level were more often done, for example working with community organizations and providing education at schools'' - (r. 398-403 of the marked manuscript)

''Many healthcare professionals described the pressure of the social norm, embedded in the community level, among women of Turkish, Moroccan, and Syrian origin as a barrier for obtaining, processing, and understanding information. For example, they described that these women experience peer pressure and blindly copy what their friends and/or family members do and say, reflecting factors at the interpersonal level.'' - (r. 426-430 of the marked manuscript)

''According to stakeholders across all categories, another barrier experienced by Turkish-, Moroccan-, and Syrian women is seeking care from a healthcare professional, such as their GP, referring to interactions at the interpersonal level. Stakeholders across all categories identified a lowered proficiency of the Dutch language as underlying cause at the individual level for this barrier, especially for the older women with a migration background. Healthcare professionals also mentioned lower health prioritization as a barrier at the individual level, particularly for younger women with a migration background. Healthcare professionals described these

younger women as a 'forgotten group' in health communication practices.'' - (r. 431-439 of the marked manuscript)

''For the older women with a migration background, stakeholders across all categories mentioned that the barrier for visiting their GP is often because of experiencing a language barrier at the individual level and the discomfort they experience at community level due to shame and taboo regarding speaking about their intimate parts related to CC screening.'' - (r. 449-452 of the marked manuscript)

''Although healthcare professionals also try their best to meet the information needs of women of Turkish, Moroccan, and Syrian origin with their demand-driven approach, they indicated to still encounter barriers in health communication practices. The main reason for this, as indicated during the FGDs, was a lack of time during their consultation hours. This occurs from a restricted consultation time determined at system level leading to constraints at communication at the interpersonal level. There is also a gap in that women of Turkish, Moroccan, and Syrian origin identified their GP as their preferred source of information regarding both CC screening and HPV vaccination, whereas described that at the system level it is defined that the GP works in a symptoms-oriented manner, meaning that patients do not visit their GP on a regular basis to discuss certain matters including prevention, but only when they have certain symptoms.'' - (r. 469-479 of the marked manuscript)

''A few healthcare professionals mentioned that they followed e-learnings and education courses to overcome barriers in health communication practices with women with a migration background and other vulnerable groups. They indicated that these materials primarily focus on interpersonal interactions related to preventive care programs, motivational conversations, and non-vaccinating individuals in general, but do not address group-specific information needs at community level.'' - (r. 495-500 of the marked manuscript marked)

Theme 3 is now stated as follows:

''Stakeholders across all categories mentioned that these informational meetings should be organized at the interpersonal level, via collaborations with key community leaders and healthcare professionals. Key community leaders, who are highly trusted at the community level, reported having valuable insight into culturally sensitive methods for health communication practices. Healthcare professionals were identified as trusted sources of information at the interpersonal level. Both stakeholder categories acknowledged the potential for mutual collaboration. Stakeholders across all categories suggested to emphasize the fact that the Islam highly prioritizes taking good care of your own body and health, reflecting a facilitator at community level.'' - (r. 518-526 of the marked manuscript)

''For the CC screening, it was mentioned that it could be helpful to support women with requesting the self-testing toolkit. Stakeholders indicated that requesting the kit is often experienced as complicated at the individual level due to the needed digital skills.'' - (r. 529-532 of the marked manuscript)

Theme 4 is now stated as follows:

''The focus was on stakeholders at the interpersonal level, namely key community figures and healthcare professionals, who were identified as important sources of information by women of Turkish, Moroccan, and Syrian origin in a previous study (3).'' - (r. 555-558 of the marked manuscript)

''To organize informational meetings, key community figures indicated the need to increase their knowledge regarding CC and its prevention programs. Stakeholders across all categories suggested that this could be done via capacity building at the community level by providing training, since many of the key community figures do not have experience with communicating information regarding CC prevention.'' - (r. 561-565 of the marked manuscript)

''Healthcare professionals indicated that they also have a need for further training at the interpersonal level, particularly in conversational techniques.'' - (r. 579-580 of the marked manuscript)

Theme 5 is now stated as follows:

''Stakeholders across all categories emphasized the similarities at community level regarding cultural and religious aspects among women of Turkish, Moroccan, and Syrian origin.'' - (r. 597-599 of the marked manuscript)

''According to the stakeholders across all categories, another aspect at the individual level that needs to be considered is the variety in language proficiency among women of Turkish, Moroccan, and Syrian origin.'' - (r. 626-628 of the marked manuscript)

''Key community figures indicated that those with higher levels of education might be more critical of the provided information, while healthcare professionals observed that decision-making among less educated individuals is often more strongly influenced at the interpersonal level via their social environment.'' - (r. 636-639 of the marked manuscript)

''They noted that in urban settings, there are often more mosques and community organizations that actively organize activities to share information at community level.'' - (r. 663-665 of the marked manuscript)

Theme 6 is now stated as follows:

''Stakeholders across all categories emphasized that health communication practices should be done at the interpersonal and community level via social networks.'' - (r. 679-680 of the manuscript incl. revisions)

''These were locations at interpersonal and community level, for example mosques, schools, community centers, and healthcare facilities.'' - (r. 705-707 of the manuscript incl. revisions)

''They mentioned that relevant alternatives for information materials, for example translations of the information brochures, are already made available from system level, but do not reach women with a migration background at the individual level adequately.'' - (r. 738-741 of the manuscript incl. revisions)

- The findings frequently combine perspectives across stakeholder categories, making it difficult to determine whether particular views were shared by all groups or expressed by only one stakeholder category. Presenting areas of convergence and divergence more explicitly would strengthen the credibility and usefulness of the findings.

Amended. Throughout the Results section where 'stakeholders' was stated, we adjusted that to 'stakeholders across all categories' to emphasize that this perspectives was shared by stakeholders from all categories. Perspectives that were specific for certain stakeholder categories are indicated with that stakeholder category in particular.

- The Results primarily emphasize areas of agreement among participants. Were any divergent or minority perspectives identified? Reporting contradictory viewpoints would provide a more balanced and nuanced interpretation.

Thank you for this suggestion. During the FGDs, stakeholders from different groups largely expressed converging views on the communication gaps and potential interventions, but explicit contradictory viewpoints were rare. We have revisited the transcripts to check for minority or divergent perspectives, but did not identify substantial opposing perspectives that would change the interpretation of our findings. We have added a statement in the Discussion to clarify that the themes primarily reflect areas of consensus across stakeholder categories and that divergent or minority viewpoints did not emerge as distinct themes *(r. 765-768 of the marked manuscript).* This is now stated as follows:

> *''Stakeholders across all categories largely converged in their descriptions of communication gaps and feasible interventions. Divergent or minority perspectives did not emerge and therefore did not form distinct themes, suggesting a shared perspectives of the main barriers and needs.''*

- Some findings are presented collectively for Turkish-, Moroccan-, and Syrian-Dutch women despite acknowledging important differences between these populations later in the Results. Consider ensuring that conclusions remain appropriately nuanced and avoid unnecessary generalization across distinct migrant communities.

Thank you for addressing this point. We agree that women of Turkish, Moroccan, and Syrian origin are distinct communities and that unnecessary generalization should be avoided. In our FGDs, however, stakeholders predominantly described communication gaps and potential interventions aimed at women of Turkish, Moroccan-, and Syrian origin, rather than proposing distinct strategies for each group. This aligns with our initial aim to explore communication approaches that could be implemented for these groups of women. At At the same time, stakeholders highlighted important differences (i.e. religious variety, varying levels of language proficiency, variations between generations, variations between urban and rural settings), which we have described in Theme 5 and emphasizedin the Discussion section to ensure that our conclusions acknowledge both the shared issues and the group-specific nuances *(r. 759-765 of the marked manuscript).* This is now stated as follows:

> *'' Stakeholders predominantly described communication gaps and potential interventions as relevant to women of Turkish-, Moroccan-, and Syrian origin collectively, and did not propose distinct strategies for each group. This is consistent with our aim to explore communication approaches that could be implemented for these groups of women. At the same time, stakeholders pointed to important differences that should be considered when tailoring communication, such as religion, language proficiency, generation of migration, and living in urban versus rural settings (see Theme 5).''*

- The Results occasionally present practical recommendations alongside empirical findings. Consider separating participants' reported perspectives from the authors' broader recommendations, reserving interpretation and implications primarily for the Discussion section.

Thank you for addressing this point. The Results section of our manuscript only presents practical recommendations that were explicitly mentioned by participants, as these formed part of the data collection during the FGDs (Themes 3 and 4). We have re-checked the Results to ensure that all recommendations are clearly attributed to the stakeholders and that broader interpretations and implications were described in the Discussion.

Discussion

- The Discussion occasionally presents stakeholders' perceptions as though they represent the actual experiences or needs of Turkish-, Moroccan-, and Syrian-Dutch women. The interpretation should consistently distinguish between stakeholders' views and evidence obtained directly from the target population. This distinction is important for maintaining interpretive validity.

Amended. We have revised the Discussion section by explicitly mentioning that findings regarding the experiences and needs of women of Turkish, Moroccan, and Syrian origin stems from stakeholders' perspectives *(r. 768-769 of the marked manuscript)*. This is now stated as follows:

> *''According to the stakeholders perspectives, the identified gaps might be attributed to misconceptions, lack of awareness, and doubts regarding the programs' effectiveness.''*

- The Discussion occasionally implies causal or effectiveness claims regarding proposed interventions. Given the exploratory qualitative design, these statements should be tempered to reflect that the study identifies perceived opportunities rather than demonstrating intervention effectiveness.

Amended. We agree that, given our exploratory qualitative design, the Discussion should not imply causal or effectiveness claims regarding the proposed interventions. We have therefore revised the wording in the Discussion section to nuance such statements. This is now stated as follows:

> *''According to the stakeholders' perspectives, the identified gaps might be attributed to misconceptions, lack of awareness, and doubts regarding the programs' effectiveness. Therefore, stakeholders across all categories highly recommend to also include stakeholders from the community level in health communication practices, rather than operating solely from the system level.'' - (r. 768-772 of the marked manuscript)*

> *''Therefore, they highlighted the need for interventions aimed at enhancing their skills in communication practices regarding CC prevention to the needs of women of Turkish, Moroccan, and Syrian origin. Strengthening these stakeholders' competencies might support women of Turkish, Moroccan, and Syrian origin in making an informed decision regarding participation in CC prevention programs.'' - (r. 775-781 of the marked manuscript)*

> *''Findings from the present study indicate that, according to stakeholders' perspectives, social support is insufficiently considered in current health communication practice. Role definitions in primary care are determined at system level, for example that the GPs should work in a symptoms-oriented focus. This does not align with the needs and expectations of women of Turkish, Moroccan, and Syrian origin who preferred their GP also for preventative health. Stakeholders indicated that the interpersonal and community level are often not actively involved, although they could play a key role in supporting IDM.'' - (r. 793-800 of the marked manuscript)*

> *''Stakeholders in this study also described their perspectives on how to better align health communication practices with the lived experiences of these women.'' - (r. 811-812 of the marked manuscript)*

> *''Stakeholders suggested that healthcare professionals could support key community figures by increasing their knowledge regarding CC and CC prevention programs. Another suggestion was that key community figures could provide healthcare professionals and other stakeholders from the system level with valuable insights into the barriers individuals face in making*

informed decisions regarding CC prevention, as well as highlight the cultural, religious, emotional, and practical aspects associated with these barriers. They could support familiarity by making health communication practices more recognizable and accessible, and fostering trust between individuals and the CC prevention programs. For example, this can be done by organizing collaborative educational sessions for key community figures and healthcare professionals, co-creation of information materials, and holding interactive events where women of Turkish, Moroccan, and Syrian origin could share their lived experiences, concerns, and ask questions. Stakeholders expressed the need for considering the variations among women of Turkish, Moroccan, and Syrian origin. Moreover, for successful implementation, they mentioned that it is recommended to align the interventions as closely as possible with the lived experiences of women of Turkish, Moroccan, and Syrian origin.'' - (r. 876-892 of the marked manuscript)

- Throughout the manuscript, consider using terminology consistent with qualitative research methodology. For example, "transferability" may be more appropriate than "generalisability" when discussing the broader applicability of qualitative findings.

Amended. We checked the manuscript for consistent use of qualitative terminology and have revised the relevant sentences accordingly. This is now stated as follows:

''This was done to identify overarching patterns and insights that may support transferability to similar contexts, but may have limited the observation of category-specific phenomena.'' - (r. 833-835 of the marked manuscript)

''The project team considered this scenario-based approach as more useful than the previous list of questions, as it allowed participants to engage with realistic and potential familiar situations and encouraged them to reflect on their own experiences and perspectives.'' - (r. 254-256 of the marked manuscript)

''Their established relationships enable successful health communication practices and address concerns or reduce fears.'' - (r. 684-685 of the marked manuscript)

''For example, key community figures share similar cultural and religious norms and values and if they are actively involved in communication practices regarding CC prevention, these aspects could be adequately incorporated.'' - (r. 851-854 of the marked manuscript)

''Such a setting might have illuminated pathways for more enhanced stakeholder engagement and intersectoral cooperation.'' - (r. 827-829 of the marked manuscript)

- Several behavioral theories (COM-B, Social Cognitive Theory, Reasoned Action Approach, Narrative Communication Theory) are introduced for the first time in the Discussion. Their relationship with the Socio-Ecological Model, which serves as the study's primary conceptual framework, should be better explained to avoid conceptual fragmentation. Alternatively, if these theories were intended to inform the study, they should be introduced earlier in the Background and reflected consistently throughout the manuscript.

Amended. The SEM was indeed our primary conceptual framework and guided the study design, stakeholders' selection, and analysis. In the Discussion, we refer to specific behavioral theories to deepen the interpretation of our findings. We have revised the Discussion section by describing how these behavioral theories complement the SEM *(r. 784-800 of the marked manuscript).*

''While the SEM was our primary conceptual framework to capture multi-level influences on IDM, specific health behavior theories, including the COM-B model (9), the Social Cognitive Theory (10), and the Reasoned Action Approach (11), emphasize that IDM depends not only on individuals' knowledge and their practical capabilities at the individual level, but also on personal motivation and self-efficacy. On the interpersonal level, social support also plays a role, indicating that in the context of CC prevention programs IDM can be understood as a multi-level process in which the individual and interpersonal level are interacting. Findings from the present study indicate that, from stakeholders' perspectives, social support is insufficiently considered in current health communication practices, as stakeholders operating at the interpersonal and community level are often not actively involved, although they could play a key role in supporting IDM.''

- The conclusion would be strengthened by focusing primarily on the study's principal findings and their implications. The description of ongoing intervention development is interesting but is not itself a finding of the present study and could be shortened or relocated.

Amended. We have shortened the description of ongoing intervention development *(r. 892-894 of the marked manuscript).* This is now stated as follows:

''Our findings informed the development of a training for key community figures and culturally sensitive videos regarding CC screening. The videos were evaluated in a qualitative and quantitative study (manuscripts in preparation).''

Reviewer 2:

Prof. Simon Manga, University of Alabama at Birmingham Health System, Cameroon Baptist Convention Health Service

<u>Comments to the Author:</u>
This manuscript addresses an important and underexplored topic on improving communication around cervical cancer screening and HPV vaccination among migrant populations through engagement of community and healthcare stakeholders. The study is timely, relevant to public health, and provides potentially actionable insights for improving IDM. The inclusion of multiple stakeholder groups and use of the socio-ecological model are notable strengths. However, there are a few minor comments that the authors should consider addressing to further strengthen the manuscript:

1. The manuscript should address saturation and reflexivity

Amended. We have included a sentence under the 'Recruitment' part in the Methods section addressing data saturation *(r. 242-244 of the marked manuscript).* This is stated as follows:

''Recruitment continued until data saturation was reached within each stakeholder category, defined as the point at which no new themes emerged during the analysis of the last FGD.''

Regarding reflexivity, we elaborated on the role of the moderator under the 'Data collection' part in the Methods section *(r. 264-269 of the marked manuscript).* This is now stated as follows:

''NHS had a good command of Modern Standard Arabic and shared similar cultural and religious backgrounds with women of Turkish, Moroccan, and Syrian origin. This facilitated

trust and openness in the FGDs with key community figures. At the same time, the research team engaged in continuous reflexivity regarding how shared background and assumptions might shape questioning, probing, and interpretation during the FGDs and subsequent data analysis.''

2. Participants recruitment through project networks and subsequently snowball sampling raises concerns of potential bias. This is because participants may already be highly engaged in health promotion activities, leading to favorable views toward community engagement interventions

Thank you for addressing this point. We acknowledge that recruitment through project networks and snowball sampling may have contributed to a sample in which participants are relatively engaged in health promotion activities. However, our sample included a broad range of stakeholders. Across all stakeholder categories, there were participants who had no prior experience in health promotion activities at community level. Furthermore, participants were not informed about the proposed community engagement interventions prior to study participation; these were introduced and discussed only during the FGDs. Therefore, pre-existing favorable views towards the interventions themselves could not have influenced their decision whether to participate in the FGD. We agree that recruitment via the project's networks and subsequent snowball sampling may have led to an over-representation of stakeholders who are more easily accessible or connected to organizations collaborating in the project. We have acknowledged this as a limitation in the Discussion (*r. 842-847 of the marked manuscript)*. This is now stated as follows:

"Our recruitment strategy, which relied on project networks and subsequent snowball sampling, may have favored the inclusion of stakeholders who were relatively engaged in promotion activities. However, our sample also included stakeholders without prior experience in health promotion activities at community level, and participants were not aware of the specific proposed interventions beforehand. This reduced the likelihood that pre-existing positive attitudes towards the interventions themselves biased recruitment.''

3. The manuscript states FGDs ranged from 5 to 14 participants. A focus group of 14 participants is relatively large and may limit participation from quieter individuals.

Thank you for addressing this point. We agree that such a group size may limit participation from quieter individuals. In this case, the larger group resulted from our decision to join an existing, regularly meeting group of key community figures. We considered it important not to exclude members of this established group, as they all played relevant roles in their communities. To mitigate the risk of limited participation, the moderator actively encouraged contributions from all participants, used targeted probing for quieter individuals, and monitored turn-taking during the discussion. We have included a justification of this larger group size in the 'Data Collection' part of the methods section in the manuscript *(r. 247-250 of the marked manuscript).* This is now stated as follows:

''FGDs ranged from 5 to 14 participants. The largest group resulted from joining an existing, regularly meeting group of key community figures, and we chose not to exclude members of this group. During all FGDs, the moderators actively encouraged contributions from quieter participants and used targeted probing to ensure that multiple voices were heard.''

4. A little more detail of the pilot FGD is required e.g. number of participants, Duration, Location

Amended. We have added more details on the pilot FGD in the 'Data Collection' part of the Methods section *(r. 256-259 of the marked manuscript).* This is stated as follows:

> *''To test the duration, set up, and design of this FGD guide, we conducted one pilot FGD with eight women of Turkish, Moroccan, and Syrian origin, face-to-face in a local community center. The pilot FGD lasted approximately 2 hours.''*

5. The moderators of the FGDs are identified only as female researchers (TV and NHS). Readers will be interested to know a little more about them e.g. academic qualification, specialty, experience

Thank you for addressing this issue. We have included a sentence under the 'Data Collection part in the Methods section with additional information regarding the professional background and qualitative research experience of the moderators *(r. 263-264 of the marked manuscript).* This is stated as follows:

> *''Both hold a Master's degree and have previous experience with conducting qualitative research and moderating FGDs.''*

References:

1. Kitzinger J. Qualitative Research: Introducing focus groups. BMJ. 1995;311(7000):299–302.
2. Krueger RA, Casey MA. Focus Groups: A Practical Guide for Applied Research: SAGE Publications; 2014.
3. Veeravagu T, Hamdiui N, Stein ML, Al-Rubaie N, van Wijk JLI, Ben Ali N, et al. Supporting Informed Decision-Making Regarding Participation in Cervical Cancer Prevention Programs: A Qualitative Study Among Turkish-, Moroccan-, and Syrian-Dutch Women. Community Health Equity Research & Policy. 2026;0(0):2752535X261427045.
4. IARC. Cancer Today: The Netherlands Fact Sheet International Agency for Research on Cancer, Lyon, France2024 [Available from: https://gco.iarc.who.int/today/en/fact-sheets-populations/528/the-netherlands.
5. Kilanowski PhD RNACFJF. Breadth of the Socio-Ecological Model. Journal of Agromedicine. 2017;22(4):295–7.
6. Jepson RG, Hewison J, Thompson AG, Weller D. How should we measure informed choice? The case of cancer screening. J Med Ethics. 2005;31(4):192–6.
7. Bolt I, Schermer MHN, Bomhof-Roordink H, Timmermans DRM. Informed Decision-Making and Capabilities in Population-based Cancer Screening. Public Health Ethics. 2022;15(3):289–300.
8. Braun V, Clarke V. Thematic analysis. 2006. p. 57–71.
9. Michie S, van Stralen MM, West R. The behaviour change wheel: a new method for characterising and designing behaviour change interventions. Implement Sci. 2011;6:42.
10. Bandura A. Social cognitive theory of self-regulation. Elsevier Science; 1991. p. 248–87.
11. Fishbein M, Ajzen I. Predicting and Changing Behavior: The Reasoned Action Approach2010.

VERSION 2 - REVIEW

**Reviewer**	**1**
**Name**	**Destaw, Alemnew**

Affiliation Mizan-Tepi University, Public health

Date 01-Oct-2026

COI

Thank you very much for the opportunity to reassess the revised version of the manuscript entitled “Stakeholders’ Perspectives on Communication Gaps in Cervical Cancer Prevention among Women of Turkish, Moroccan, and Syrian Origin: A Focus Group Study in the Netherlands.”

I have carefully reviewed the revised manuscript and am fully satisfied with the authors’ responses to my previous comments and the amendments made. The authors have adequately addressed my concerns, and I have no additional comments or concerns at this stage.

I would only suggest a final language edit to further improve the readability and clarity of the manuscript. Subject to this minor language editing, I recommend acceptance of the manuscript for publication.

I would like to appreciate the authors for carefully considering my previous comments and making the necessary revisions.

Thank you again for the opportunity to review this work.
