# Supplementary material for: Stakeholders’ perspectives on communication gaps in cervical cancer prevention among women of Turkish, Moroccan and Syrian origin: a focus group study in the Netherlands
Source: BMJ Open. 2026 Oct 20;16(10):e120419. doi: 10.1136/bmjopen-2026-120419 (PMC13684154; doi:10.1136/bmjopen-2026-120419)
Supplement: Supplementary file 2 [file bmjopen-16-10-s001.docx]

**Focus group discussion guide**

**Originally written in Dutch and translated into English for publication. The original Dutch version is available upon request from the corresponding author*

**INTRODUCTION**

Welcome everyone, I am glad that you could all be here today. First of all, I would like to thank you for participating in this group discussion. Today, we will talk together about how information regarding cervical cancer (CC) screening, also known as the smear-test or the self-test, and the Human Papillomavirus (HPV) vaccination is communicated to Turkish-, Moroccan-, and Syrian-Dutch women.

Before we continue, I would like to start with a brief round of introductions. Please tell us, in two short sentences, who you are and where you work. I will start: my name is Tharsini Veeravagu and I am a researcher at the National Institute for Public Health and Environment (RIVM).

**AGENDA**

Thank you all very much. First, I will give you an introduction about the project we are working on and why we are here together today. Then, we will discuss several scenarios that I will describe. After that, I will present you with a number of statements so we can think more deeply about possible solutions. Finally, I will give a short presentation about the results from the interviews we conducted with Turkish-, Moroccan-, and Syrian-Dutch women.

The program:

1. Introduction	(15 min)
2. Part 1: Brainstorming based on scenarios	(30 min)
3. Summarizing/Concluding possible solutions	(15 min)

Break	(10 min)

4. Part 2: Statements with possible solutions	(30 min)
5. Part 3: Results of the interview study	(15 min)
6. Closing	(5 min)

**The IMAGINE project**

Purpose of the project = To help women making an informed decision about whether or not to participate in the CC screening program (smear test/self-test) and the HPV vaccination for their children.

Target group = Turkish, Moroccan, and Syrian-Dutch women

- Aged between 30 and 60 years and/or
- With a daughter between 9 and 26 years old

We focus on these age groups because these are the groups invited to participate in the cervical screening program (smear test/self-test) and the HPV vaccination.

How = By ensuring that the information about the CC screening program (smear test/self-test) and the HPV vaccination better matches the needs of these women.

From April to June last year, we conducted interviews with women from the target group. This provided us a good understanding of their needs. I will share the results of these interviews with you at the end of the session.
Today, we would like to talk with you because we are very interested in your ideas on how the information regarding the CC screening program (smear test/self-test) and the HPV vaccination can be tailored to this target group, and how you see your own role in this process. We will use your ideas and suggestions to develop something together with the target group that will support them in making an informed decision about whether or not to participate.

We will conduct these discussions with three different groups:

1. Key community figures
2. GP, GP assistants, & Youth healthcare professionals
3. Communication experts, policy makers, and project managers

Before we start, I’d like to share some important points:

- Anything that comes to mind may be important and you are welcome to mention it. There are no right or wrong answers; we want to hear every opinion or idea.
- The session will last approximately two hours.
- We would like to record the conversation so that we can use all the information accurately. The recording will be transcribed, and your name and other personal details will be omitted. The audio will be deleted immediately after transcription. Everything you say will be treated confidentially.
- Please try not to talk over one another. Listen carefully and wait until someone has finished speaking before responding. This way, we can have a respectful conversation and ensure we don’t miss any information when listening back and transcribing the discussion.

Now we can begin with the group interview. Do you have any questions before we start?

*> Answer eventual questions*

Would you prefer to address each other formally (using “you”/“sir/madam”) or informally (using first names)?
I will now start the recording.

*> Start recording.*

**PART 1: Brainstorm based on scenarios (35 min)**

I will now describe a situation, and based on this, I would like to ask you to discuss whether you recognize this, what your experiences are, and what you think could help in such cases.

**1. Scenarios for focus group discussion (FGD) with key community figures**

Situation A:

There is a friend you know from the neighborhood or the mosque. She was born and raised in Turkey. She is learning the Dutch language better and better, but government letters are still often difficult to read for her. Occasionally, you help her with this. This time, she comes to you with the invitation letter for the CC screening program. She says that she already knows it is about an examination of the uterus, but she does not really understand why she is invited for this. Therefore, she wonders whether it is really necessary to participate.

- Has anyone ever experienced a similar situation? Could you tell us more about this?
  - How did you handle it at the time?
- If not, how would you approach this? Why?
- What is it like for you to have conversations about this? Why?
- To what extent does culture play a role here? Why?
  - How can this be taken into account in communication?
  - Could something or someone help with this? If so, who and in what way(s)?
- What could help these women in making their decision whether or not to participate in the CC screening (Pap smear/self-sampling test)?
  - Who could play a role in this? Why and in what way(s)?
  - At what time/place could we do this? Why?
    - Are such topics suitable to be discussed in a community center/mosque? Why or why not?
- How could we reach and inform the largest group of women as possible about the CC screening program?
  - What should we take into account? And what is the best way to do that?
  - How can we ensure that this target group feels addressed?

*→ Briefly summarize what has been said and ask whether they agree and/or have any additions.*

Situation B:

A Syrian-Dutch woman has a 10-year-old daughter. From many mothers of her daughter's classmates, she hears that they have had their children vaccinated against HPV. She told her husband about this, as she thinks it would also be a good idea for their daughter to protect her against possible diseases in the future. Her partner disagreed, because their daughter is not yet sexually active and, according to their religious norms and values, she will not be sexually active before marriage. The mother would still like to protect her daughter, considering that her future partner may have had sex before marriage. Opinions regarding the HPV vaccination are also divided within her group of friends. She would like to get advice on how she can discuss this with people around her, even though they may have different views.

- Has anyone ever had to give advice to someone in a similar situation? Can you tell us more about this?
  - What kind of advice did you gave? Why?
- If not, what advice would you give to the woman in this example? Why?
- What could help her to start the conversation about this with people around her?
  - Who could support her with this? Why and in what way(s)?
- How could we reach as many people as possible with information about HPV vaccination for their daughters/children?
  - What should we take into account? And what is the best way to do this?
  - How do we ensure that this target group feels addressed?

*→ Briefly summarize what has been said and ask whether they recognize themselves in this and/or have any additions.*

**2. Scenarios for FGD with general practitioners (GPs), GP assistants, and Youth Health Professionals**

Situation A:

A Moroccan-Dutch woman comes to your consultation with vague complaints, including lower abdominal pain. She is 45 years old, so during the conversation, you ask if she has ever had a Pap smear. She indicates that she is not familiar with this and says she has never received an invitation for it. During your brief explanation about the CC screening program, you notice that she becomes uncomfortable while discussing this topic.

- Has anyone ever encountered a similar situation? Could you tell us more about this?
  - How did you handle it at the time? Why?
- If not, how would you approach this? Why?
- What is it like for you to have conversations about this? Why?
  - What could help you in such conversations? And what would not help? Why?
- In your opinion, what should women in situations like this know about the CC screening program? Why?
- How can we ensure that these women are able to make an informed decision about participating in the CC screening program?
  - Are there other people, besides yourself, who could play a role in this? Why and in what way(s)?
  - What are appropriate moments/places for this, and what are not? Why?
    - For Youth Health professionals: Do you see a role for yourself and/or your organization in providing information about the CC screening program? Why or why not?
      - In what way(s) could this be done?
      - What do you need for this?
- How could we reach and inform as many women as possible about the cervical cancer screening program?
  - What should be taken into account? And what is the best way to do this?

*→ Briefly summarize what has been said and ask whether they agree and/or have any additions.*

Situation B:

A Syrian-Dutch woman has a 10-year-old daughter. You tell her that her daughter is eligible for the HPV vaccination. The woman has heard many negative stories from people around her and is afraid of side effects. She does not think it is necessary, because her daughter is not yet sexually active and, according to their religious norms and values, she will be sexually active before marriage. She also hears from many other mothers that they have not vaccinated their children against HPV.

- Has anyone ever experienced a similar situation? Could you tell us more about this?
  - How did you handle it at the time? Why?
  - How did the woman respond?
- If not, how would you approach this? Why?
- How can we ensure that women receive the correct information?
  - Who could play a role in this? Why and in what way(s)?
    - To GPs/GP assistants: Do you see a role for yourself and/or your organization in providing information about the HPV vaccination? Why or why not?
      - In what way(s) could this be done?
      - What would you need for this?
- How could we reach as many people as possible regarding the HPV vaccination for their daughters/children?
  - What should we take into account? And what is the best way to do this?

*→ Briefly summarize what has been said and ask whether they agree and/or have any additions.*

**2. Scenarios for FGD with communication experts, policy makers, and project managers**

A Turkish-Dutch woman receives an invitation for the CC screening program and an invitation for her 10-year-old daughter for the HPV vaccination. She has difficulty understanding the information leaflets and is unsure why participation is necessary. She is unable to find a digital translation, and she does not feel comfortable asking people around her for help, given the sensitive nature of the topic.

- In your opinion, what should women in situations like this know about the CC screening program? Why?
  - And regarding the HPV vaccination? Why?
- How could we make it easier for this woman to understand the information?
  - What tools could help her? Why?
    - Through which other means, besides text, could the information be communicated? Why?

*→ Briefly summarize what has been said and ask whether the participants agree and/or have any additions.*

**WRAPPING UP PART 1: Posible solutions (15 min.)**

We have just discussed several situations concerning women making decisions about whether or not to participate in the CC screening program (Pap smear/self-sampling) and whether or not to have their children vaccinated against HPV.

- In your opinion, what could help ensure that these women are better informed about the CC screening program (Pap smear/self-sampling) and the HPV vaccination?
- How can we best implement this?
  - What do we need? Why?
  - Who do we need? Why?
  - Where should we do this? Why?

*In the FGD with communication experts, policy makers, and project managers we will also present the ideas that were suggested by key community figures and healthcare professionals:

- Podcast for healthcare providers about cultural aspects in the decision-making process
- Training for key figures to provide education sessions
- Information sessions/meetings (in the women’s own language)
- Involving experts-by-experience
- Videos (representative of the target group)
- Invitation through the gGP
- More information about the contents of the vaccine (addressing distrust)
- What do you think of the suggestions from the key community figures and healthcare professionals?
  - What are the advantages and disadvantages? Why?
- Do you think these could be effective ways to reach and inform women about the CC screening program? Why or why not?
  - And about the HPV vaccination? Why or why not?
- Is it feasible to implement this on a larger scale? Why or why not?
  - What would be needed for this? Why?
  - With whom should or could we collaborate to achieve this? Why and in what way(s)?

Is there anyone who would like to add something to what we have just discussed?

Thank you very much for your input. I think now is a good time for a short break. You have about 5 to 6 minutes to do something for yourself or, for example, to go to the restroom.

***** PAUSE *****

**(10 min)**

**PART 2: statements for possible solutions (30 min)**

Welcome back, everyone. We just discussed possible solutions to better tailor the information regarding the CC screening program and HPV vaccination to the needs of the target population.

**1. Statements for FGD with key community figures**

In the previous session with key community figures, organizing informational meetings about the CC screening program and HPV vaccination was mentioned as a main suggestion. One of the proposals was to train or educate key community figures so they can provide such informational sessions themselves.

- What are your thoughts on this? Why?
- What should the content of this training be? Why?
- In what way or format could this be organized? Why?

I would now like to present several suggestions mentioned in the previous session. You can indicate (using the “raise hand” function in Teams) if you think this would help key community figures to give information sessions. If you think it would not be helpful, or that they would not use it, please do not raise your hand.

- Training/course day(s) for key community figures to provide informational sessions
- Where? Why?
- For whom? Why?
- By whom? Why?
- Where/how often? Why?

*→ Briefly summarize what was said and ask if they agree with this or have any additions.*

- E-learning? Why or why not?
- When/how often? Why?
- What should this include? Why?

*→ Briefly summarize what was said and ask if they agree with this or have any additions.*

- Podcast? Why or why not?
- In what way/with what content? Why?
- What type of stories should be included? Why?
- For whom? Why?

*→ Briefly summarize what was said and ask if they agree with this or have any additions.*

As part of the informational sessions:

- Videos? Why or why not?
- In what way/with what content? Why?
- Animation or real-life? Why?
- Where should they be shown? Why?

*→ Briefly summarize what was said and ask if they agree with this or have any additions.*

- Leaflets? Why or why not?
- In what way/with what content? Why?
- Where should they be distributed? Why?

*→ Briefly summarize what was said and ask if they agree with this or have any additions.*

**2. Statements for FGD with GPs, GP assistants, and Youth Health Professionals**

During the previous discussion with healthcare providers, it was mentioned, among other things, that there is a need for greater insight into the cultural and religious aspects that play a role for this target group in making decisions regarding participation in the CC screening program and HPV vaccination. One of the suggestions was to provide additional training for healthcare providers on these aspects.

- What are your thoughts on this? Why?
- What content should this training include? Why?
- In what way or format could this be organized? Why?

I would now like to present several suggestions that were mentioned in the previous discussion. You may indicate (using the “raise hand” function at the top of the Teams screen) if you think this would help healthcare providers gain more insight into the cultural and religious aspects. If you think it would not be helpful or that they would not use it, please do not raise your hand.

- Organizing a meeting/symposium? Why or why not?
- Where? Why?
- When/how often? Why?
- For whom? Why?

*→ Briefly summarize what was said and ask if they agree or have any additions.*

- E-learning? Why or why not?
- When/how often? Why?
- What should it be about? Why?

*→ Briefly summarize what was said and ask if they agree or have any additions.*

- Podcast? Why or why not?
- What kind of story/stories should be included? Why?
- For whom? Why?
- By/with whom? Why?

*→ Briefly summarize what was said and ask if they agree or have any additions.*

- Visual conversation aids for use during consultations? Why or why not?
- What should be included? Why?
- For what purpose would these be used? Why?

*→ Briefly summarize what was said and ask if they agree or have any additions.*

**3. Statements for FGD with communication experts, policy makers, and program managers**

I would now like to present several suggestions that we have gathered from the previous FGD with key community figures and healthcare professionals. You can indicate (using the “raise hand” function at the top of the Teams screen) if you think this would help to better inform the target group. If you think it would not help them sufficiently in making their decision, please do not raise your hand.

- Organizing information meetings? Why or why not?
- In what way/with what content? Why?
- Where? Why?
- For whom? Why?
- Who do you think should provide the information? Why?

*→ Briefly summarize what was said and ask if they agree or have any additions.*

- Training/course for key figures to provide information sessions
- In what way/with what content? Why?
- How should this training be organized? Why?
- Who else could benefit from such training? Why?

*→ Briefly summarize what was said and ask if they agree or have any additions.*

- Additional training for healthcare providers with this specific target group as patients
- For whom could this be useful? Why?
- What content is necessary? Why?
- How/in what way should this training be organized? Why?

*→ Briefly summarize what was said and ask if they agree or have any additions.*

- E-learning? Why or why not?
- When/how often? Why?
- What should the content include? Why?

*→ Briefly summarize what was said and ask if they agree or have any additions.*

- Podcast? Why or why not?
- In what way/with what content? Why?
- What kind of story/stories should be included? Why?
- For whom? Why?

*→ Briefly summarize what was said and ask if they agree or have any additions.*

- Visual conversation aids for use during consultations? Why or why not?
- What should be included? Why?
- For what purpose would these be used? Why?

*→ Briefly summarize what was said and ask if they agree or have any additions.*

- Videos? Why or why not?
- In what way/with what content? Why?
- Animation or real-life? Why?
- Where should they be shown? Why?

*→ Briefly summarize what was said and ask if they agree or have any additions.*

- Leaflets? Why or why not?
- In what way/with what content? Why?
- Where should they be distributed? Why?

*→ Briefly summarize what was said and ask if they agree or have any additions.*

Other suggestions from previous discussions:

- Invitation via the general practitioner
- More information about the contents of the vaccine (due to mistrust)
- What do you think of the suggestions from the key figures and healthcare professionals?
  - What are the advantages and disadvantages? Why?
- Could these be effective ways to reach and inform women about the cervical cancer screening programme? Why or why not?
  - And about the HPV vaccination? Why or why not?

Is there anyone who would like to add anything to what we have just discussed? Nora/Nadien, do you have any additions or questions for the group?

We will now move on to the final part of today’s session, the interview results.

**PART 3: Results previous interview study with target population (15 min)**

We have now spent considerable time discussing and reflecting on possible ways to better inform women about cervical screening/self-sampling at home and the HPV vaccination. As I mentioned at the beginning, we have also discussed these topics with the women themselves. I will now briefly present what we heard during these conversations, after which we can discuss together to what extent this aligns with what we have discussed today, as well as what stands out or may be missing.

I will do this in three parts: first, regarding the content of the information; second, through which sources they would prefer to receive this information; and finally, in what way they would prefer this information to be provided.

Content of the information

- Topics identified:
- Why should you participate?
- Causes, symptoms, etc. of cervical cancer
- What does the screening process involve? (What are the steps?)
- How likely am I to develop cervical cancer?
- What are the possible disadvantages or risks of participating?
- Emphasize alternative options for screening, such as requesting a self-sampling kit for home use and/or the possibility to request a female assistant to perform the screening.
- What can I do myself to prevent the disease?
- Discussion questions:
- What are your thoughts on these topics, and why?
- What stands out to you about these topics, and why?
  - Which of these do you recognize?
  - Which ones surprise you?
  - Which topics do you think are missing?

*→ Briefly summarize what was said and ask if they agree or have any additional comments.*

Sources of information

We also asked the women through which sources they would prefer to receive this information.

- The most frequently mentioned sources were: organizing information meetings, audiovisual materials, and the general practitioner.
- Information leaflets and the internet (with the RIVM as sender) were also mentioned often.
- In the interviews, women also indicated that they would consider using a telephone helpline, contacting the Public Health Service (GGD), or consulting a friend or family member with a medical background. When we asked further, they mentioned that they had not used these options before, but would appreciate having them available.
- Other sources mentioned several times included receiving information via family, friends or acquaintances, a theater performance, and apps.
- One notable suggestion was to include this information in the civic integration course.

Discussion questions:

- What are your thoughts on these sources, and why?
- What stands out to you about these sources, and why?
  - Which of these surprise you?
  - Which do you think are missing?
- Which sources do you consider most suitable for providing information to our target group, and why?
  - And which are the least suitable? Why?

*→ Briefly summarize what was said and ask if they agree or have any additional comments.*

Preferences for receiving information

Finally, regarding the preferences for how to receive information:

- The most frequently mentioned preference was the need for accurate translations.
- Many women also indicated that they prefer to have conversations about these topics and to be able to ask questions.
- They expressed a desire to be told where they can find the information.
- They would also like the opportunity to look up this information themselves.
- There is also a need for more information about the practical, emotional, religious, and cultural aspects of the cervical cancer screening program and the HPV vaccination.

Discussion questions:

- What are your thoughts on these preferences, and why?
- What stands out to you about these preferences, and why?
  - Which of these surprise you?
  - Which do you think are missing?
- Which preferences do you consider most suitable for providing information to our target group, and why?
  - And which are the least suitable? Why?

*→ Briefly summarize what was said and ask if they agree or have any additional comments.*

**CLOSING (5 min)**

I would like to thank you all for your time and attention today. Before we conclude, I would like to ask each of you to think of maximum three words that, in your opinion, are essential to better inform women from this target group about cervical screening/self-sampling at home and the HPV vaccination.

Go around the group and ask everyone to share their words.

- Are there others you think we should speak with about this topic, as we have done today? Why or why not?
  - What do you think they could contribute? Why?

Thank you very much. I will now stop the recording.

*> Stop the recording.*

- How did you experience today’s discussion?
- Does anyone have any other questions? (For example, about the rest of the project)

I would like to thank you once again for your participation. Your input has been very valuable for our research. If you have any further questions or comments, please feel free to send us an email.
Once we have completed all the sessions, we will work with all the ideas and suggestions we have collected. It is possible that we may contact you again in the future to ask for your opinion.
As a token of appreciation for your participation today, you will receive a €20 gift card. I wish you all a pleasant day/evening!
