# Supplementary material for: Stakeholders’ perspectives on communication gaps in cervical cancer prevention among women of Turkish, Moroccan and Syrian origin: a focus group study in the Netherlands
Source: BMJ Open. 2026 Oct 20;16(10):e120419. doi: 10.1136/bmjopen-2026-120419 (PMC13684154; doi:10.1136/bmjopen-2026-120419)
Supplement: Supplementary file 3 [file bmjopen-16-10-s002.docx]

**Short questionnaire for stakeholders participating in the focus group discussions**

**Originally written in Dutch and translated into English for publication. The original Dutch version is available upon request from the corresponding author*

**Name:……………………………………………….**

**I am…….**

- Woman
- Man
- Non-binary
- I would rather not say

**How would you describe your job/function?:**

- Key community figure
- Employee at a foundation/organization
  - Name/description:……………………………………………………………..
- Volunteer at a community center
- Volunteer at a mosque
- General practitioner
- General practitioner assistant
- Youth health physician
- Youth health nurse
- Youth healthcare professional
- Communication expert
- Policy maker
- Program manager
- Other, namely: ……………………………………………………………………………..

**How many years of work experience do you have in this current job/function?**

……………………

**What are the first two digits of the postal code of your current workplace**

……………………
