# Supplementary material for: Stakeholders’ perspectives on communication gaps in cervical cancer prevention among women of Turkish, Moroccan and Syrian origin: a focus group study in the Netherlands
Source: BMJ Open. 2026 Oct 20;16(10):e120419. doi: 10.1136/bmjopen-2026-120419 (PMC13684154; doi:10.1136/bmjopen-2026-120419)
Supplement: Supplementary file 4 [file bmjopen-16-10-s003.docx]

**Informed consent forms for stakeholders participating in the focus group discussions**

**Originally written in Dutch and translated into English for publication. The original Dutch version is available upon request from the corresponding author*

**Group discussion about the communication practices regarding population-based cervical cancer screening and the HPV vaccination among women of Turkish, Moroccan, and Syrian origin**

Thank you for being interested to participate in this group discussion. Prior to the start, we would ask you to sign this informed consent form. You can read more information regarding this research project in the information letter. Please carefully read the information below before you move on.

I have read the information letter. I understand this letter well.

It was possible to ask questions. I understand that, for further information, questions, or comments, I can contact the researcher Tharsini Veeravagu. Her contact details are provided in the information letter.

Participating in this group discussion is voluntary. I know I can quit at any moment and do not have to provide any reason for it.

I know that the group discussion will be audio-recorded, and I have no objection to that. After finalizing the transcription, the audio-recording will be directly removed. The transcript will be stored for **maximum 10 years** after finalizing the project, in a secured environment of the RIVM.

If I would like to participate in a next study, my contact details (e-mail and/or telephone number) will be stored for **maximum 5 years** after finalizing the project. If I would not like to participate in a next study, these will be removed directly after finalizing the project.

My answers could be used for (scientific) articles. This will be without mentioning my name and other personal data.

My personal data will not be shared with other organizations or persons (with the exception of the researchers from the RIVM involved in this research project) without my permission.

I can ask to remove my contact details deleted at any time. To do so, I can contact the researcher Tharsini Veeravagu. Her contact details are provided in the information letter.

Please indicate below to which you give consent to:

- I understand the text above and would like to participate in the group discussion.
- I give consent to the RIVM to contact me for a next study (please share your contact details below)

**Contact details:**

(Only fill in if you give the RIVM consent to contact you for a next study):

Telephone number:

……………………………………………………………………..

E-mail:

………………..………………..…………………………………

Name: …………………………………………………………………………..

Date: …………………………. Signature: …...………………………………….
